# Diagnosis and management of complicated urogenital schistosomiasis: a systematic review of the literature

**DOI:** 10.1007/s15010-023-02060-5

**Published:** 2023-07-19

**Authors:** Tommaso Manciulli, Davide Marangoni, Joaquin Salas-Coronas, Cristina Bocanegra, Joachim Richter, Federico Gobbi, Leonardo Motta, Andrea Minervini, Alessandro Bartoloni, Lorenzo Zammarchi, on behalf of TropNet Schisto Task Force

**Affiliations:** 1 Department of Experimental and Clinical Medicine, University of Florence, Florence, Italy; 2 Tropical Medicine Unit, Hospital Universitario Poniente, Almería, Spain; 3 Tropical Medicine and International Health Unit Vall d’Hebron-Drassanes, Infectious Diseases Department, Vall d’Hebron University Hospital, PROSICS Barcelona, Barcelona, Spain; 4 Institute of Tropical Medicine and International Health, Charité Universitätsmedizin, Corporate Member of Free University and Humboldt University Berlin and Berlin Health Institute, Berlin, Germany; 5 Infectious-Tropical Diseases and Microbiology Department, IRCCS Ospedale Sacro Cuore Don Calabria, Negrar di Valpolicella, Verona, Italy; 6 Unit of Oncologic Minimally-Invasive Urology and Andrology, Azienda Ospedaliero Universitaria Careggi, Florence, Italy; 7 Department of Infectious and Tropical Diseases, Azienda Ospedaliero Universitaria Careggi, Largo Giovanni Alessandro Brambilla, 3, 50134 Florence, Italy

**Keywords:** Praziquantel, Guidelines, Neglected tropical diseases, Hydronephrosis, Bladder cancer, Ultrasound

## Abstract

**Background:**

Currently, there are no standardized guidelines for the diagnosis or management of the complications of urogenital schistosomiasis (UGS). This systematic review of the literature aims to investigate the state of the art in reference to diagnostic approaches and the clinical management of this condition.

**Methods:**

A systematic review of literature published between January 1990 and January 2021 was conducted in the MEDLINE database, scoping for articles regarding diagnostic means or therapeutic options for the complications of UGS, namely obstructive uropathy, bladder cancer, abortion, ectopic pregnancy, infertility, kidney failure, urolithiasis and the need for invasive procedures. Relevant data were then extracted from the articles deemed eligible according to the inclusion criteria.

**Main results:**

In total, 3052 articles were identified by the research query, of which 167 articles fulfilling inclusion criteria after title/abstract screening and full-text evaluation were included, 35% on both diagnostic and therapeutic aspects, and 51% on diagnosis and 14% on therapy. Ultrasound was the most frequently tool employed for the diagnosis of UGS complications showing a good performance. Concerning the management of hydronephrosis, the majority of available evidences came from community-based studies where universal treatment with praziquantel was used leading to decrease of prevalence of obstructive uropathy. Concerning studies on surgical procedures, laser endoureterotomy followed by stenting was mostly employed in adult patients leading to a crude cure rate of 60% (43 of 71 patients). In the case of severe hydronephrosis, surgery consisting of ureteral re-implantation showed excellent results with a crude cure rate of 98% (157 cured patients of 160 treated). Concerning bladder cancer, data on 93 patients with a clear diagnosis of UGS-related bladder were available reporting a variable and sometime combined approach based on disease stage. Available data on diagnosis and management of abortion, ectopic pregnancy, infertility, kidney failure, urolithiasis and the need for invasive procedures due to UGS are also presented.

**Conclusions:**

The review produced a complete picture of the diagnostic and therapeutic options currently available for complicated UGS. These results can be useful both for guiding clinicians towards correct management and for tracing the direction of future research.

**Supplementary Information:**

The online version contains supplementary material available at 10.1007/s15010-023-02060-5.

## Background

Schistosomiasis is a parasitic neglected tropical disease (NTD) caused by trematodes belonging to the genus *Schistosoma*. There are two main clinical forms of the disease, the gastrointestinal and the urogenital. Urogenital schistosomiasis (UGS) is caused by *Schistosoma haematobium*, mostly endemic in Africa and the Middle East [1]. *S. haematobium* is prone to hybridization with several zoonotic *Schistosoma* species and this feature may have important ecological, diagnostic and therapeutic implications, currently not completely understood [2]. *S. haematobium* globally affects 112 million subjects of which 90% in Sub-Saharan Africa [3]; however, the disease represents a relevant issue also in non-endemic countries since it may affect migrants and travellers returning from endemic countries [4, 5]. Recently, some foci of autochthonous transmission have been identified in the Mediterranean area in Almería, Spain and in Corsica, France, the latter due to a *S. haematobium/S. bovis* hybrid species [6–9].

*S. haematobium* adult worms reside in the peri-vesical venous plexus. Eggs laid by females must reach the urinary tract to be released in the environment with urine and perpetuate the parasite transmission cycle. However, a proportion of eggs is trapped in the tissues of urogenital organs (e.g. bladder wall), and elicits a granulomatous inflammatory reaction followed by fibrosis [10]. Due to the consequences of this chronic inflammation, between 3.5% and 20% of affected subjects develops urogenital complications which include hydronephrosis leading to kidney failure, urolithiasis, ectopic pregnancy, infertility and bladder cancer [11, 12]. The latter is estimated to be responsible of 13,300 death per year [3].

Complicated urogenital schistosomiasis (cUGS) is potentially extremely harmful and carries a significant health burden for patients and health systems in both endemic and non-endemic areas [12, 13]. Its management is complex and often not addressed by international guidelines [14].

In high-resource countries, the management of cUGS may be suboptimal due to low awareness of health care workers in non-endemic setting leading to diagnostic delay and unnecessary use of invasive procedures [15, 16].

A systematic review of the literature was conducted to assess the current level of evidence on cUGS diagnosis and management within the activities of the TropNet Schisto Task Force (http://tropnet.eu/). Two main questions pertaining to the clinical management of cUGS were investigated: (i) What are the diagnostic methods and their performance for cUGS in endemic and non-endemic settings? (ii) What are the treatment strategies and their performance for cUGS in endemic and non-endemic settings?

## Materials and methods

**Fig. 1:**
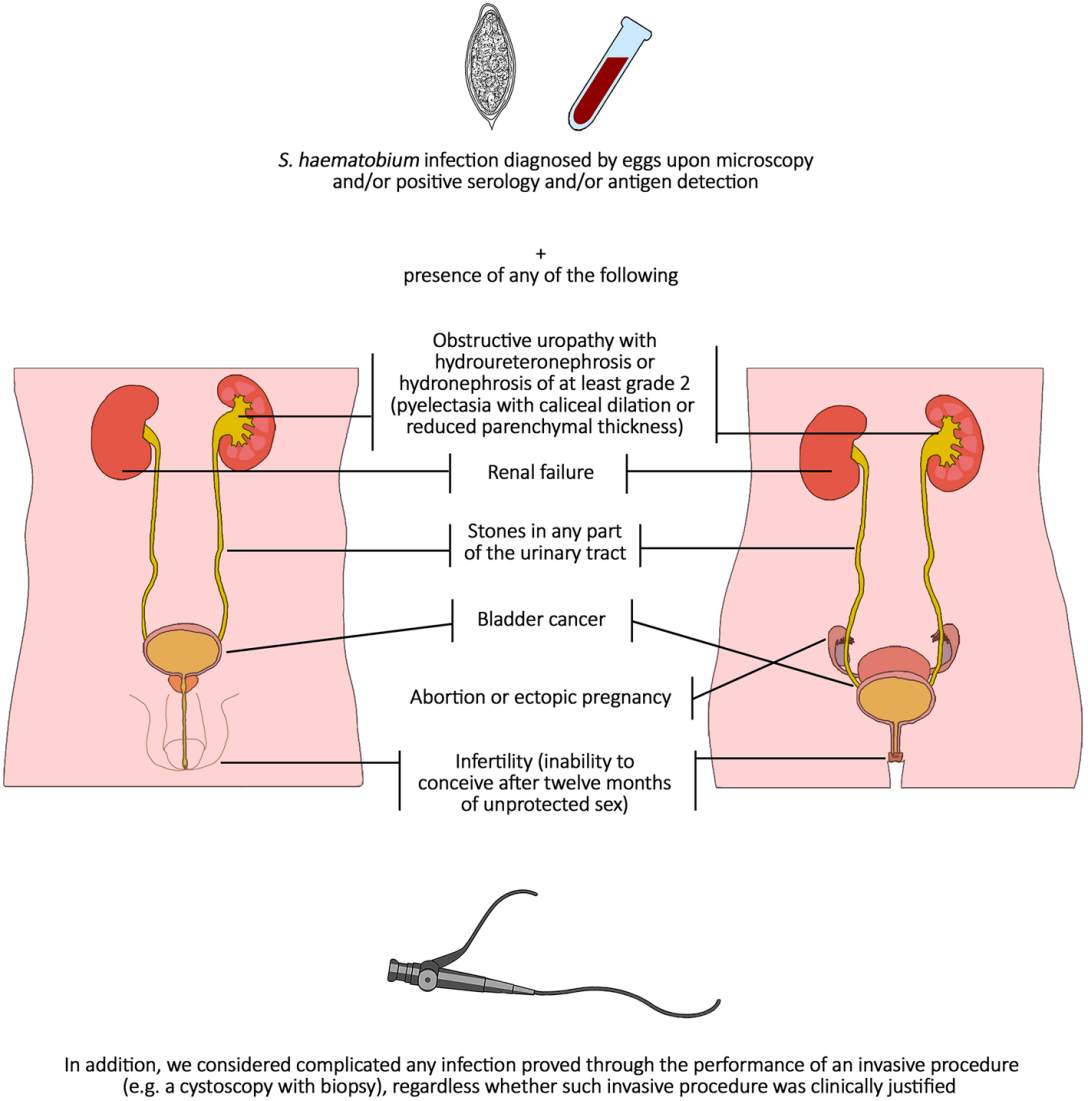
Definition of complicated urogenital schistosomiasis

Definitions of cUGS used for this review are provided in Fig. 1. The search strategy is available in the Supplementary materials and methods.

### Inclusion criteria

Inclusion criteria were:

- Papers written in Italian, English, French or Portuguese.
- Case reports, case series, clinical trials, retrospective, prospective and cross-sectional studies reporting original data on the diagnosis and/or clinical management of patients satisfying the definition cUGS.

### Exclusion criteria

Narrative and systematic reviews, animal studies, laboratory studies, studies dealing with a species different from *S. haematobium,* editorials, news reports or articles only reporting radiological data (e.g. pictorial essays) or only data on pathological anatomical aspects were excluded. Potentially eligible papers for which full text was not available and the abstract, when available, did not convey the needed information, were also excluded.

### Selection process

Two authors (T.M. and D.M.) conducted an initial independent assessment of the articles by title and abstract screening, followed by evaluation of the full texts. If doubts over eligibility were present, a third author (L.Z.) reviewed the publications to reach a collegial decision. Data extracted can be found in Supplementary Materials and Methods. For studies other than case reports or case series, data are presented as summaries of findings using Effect Direction Plots [17], grouped by paper focus (diagnostic or treatment) and by treatment category. Study type, effect direction of interventions on outcomes, differences between interventions or from baseline (together with statistical significance when reported), and sample size were visually plotted to provide an overall appraisal of the extracted data quality, characteristics and heterogeneity. No meta-analysis and formal quality assessment of extracted data were planned, expecting most articles to be case report or case series.

## Results

### Article type, setting, populations

**Fig. 2:**
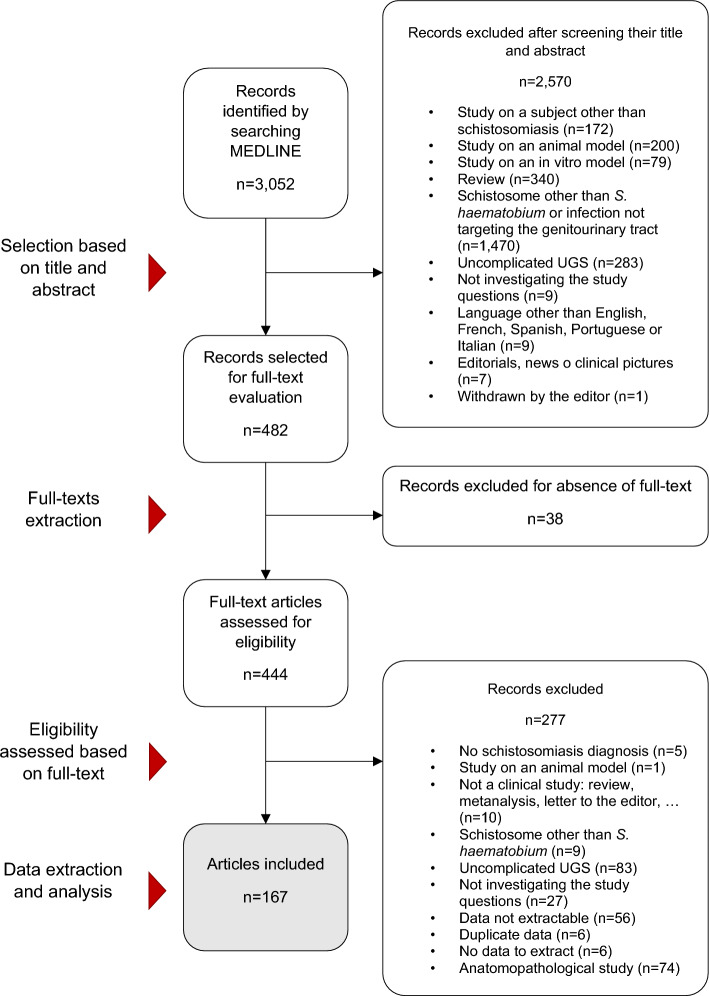
Flowchart of the literature search and selection of studies

Figure 2 shows search and selection results. Of the initial retrieved references (*n* = 3052), 167 were included in the review. Eighty-six (51.5%) articles dealt with diagnostic aspects only, 58 (34.7%) dealt with both diagnostic and therapeutic aspects and 23 (13.8%) dealt with therapeutic aspects only. Amongst 144 studies dealing with diagnostic aspects, 60 (41.7%) were carried out in centres in non-endemic areas, 77 (53.5%) were conducted in Africa and 7 (4.9%) in the Middle East. Data on the diagnosis of cUGS were available from 8,093 patients; of these patients, 7997 (98.8%) were residents in endemic areas, 49 (0.6%) were travellers, 45 (0.6%) were migrants and 2 (0.1%) were expatriates.

In studies on treatment, 3648 patients were found to have information relevant to the review for any of the cUGS clinical manifestations. Amongst 81 studies dealing with diagnostic aspects, 34 (42.0%) were carried out in centres in non-endemic areas, 41 (50.6%) were conducted in Africa and 6 (7.4%) in the Middle East. Data on the treatment of cUGS were available for 3648 patients, 3564 (97.7%) of whom were residents in endemic areas, 48 (1.3%) were travellers, 36 (1.0%) were migrants and 1 (0.1%) was an expatriate.

**Fig. 3:**
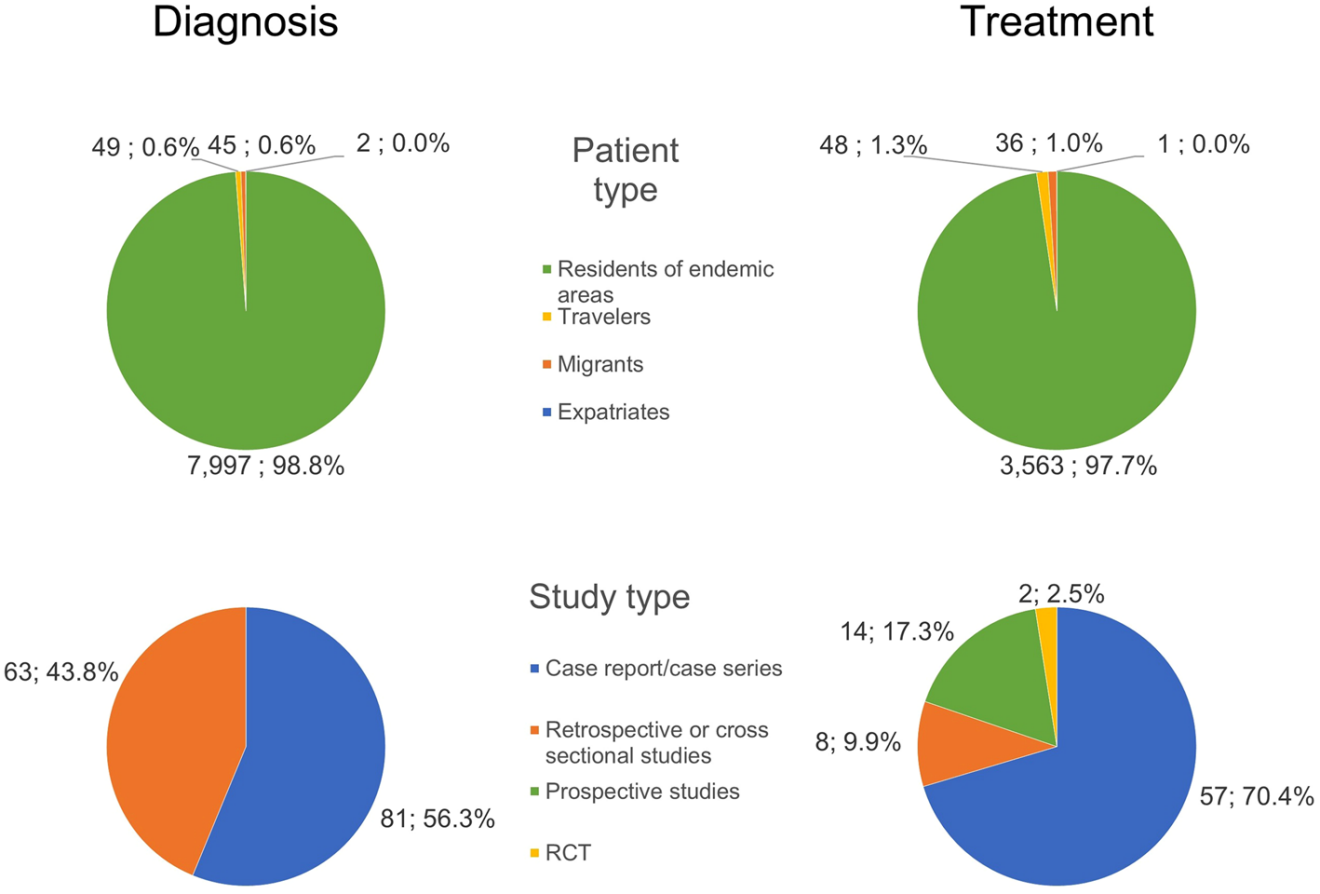
Overview of the studies and patient types included in this review

Most articles were case reports or case series (*n* = 81, 56.2% and *n* = 57, 70.4% respectively), followed by retrospective or transversal studies (*n* = 63, 43.8% and *n* = 8, 9.9%). Prospective studies were present only in the treatment group (*n* = 14, 17.3%) and only two randomized clinical trials were retrieved in the treatment group (2.5%). Figure 3 summarizes data on study and patient type included in this review, whilst Table 1 shows the different complications described in the included papers. As shown by the data presented in Tables 2 and 3 and in the Supplementary tables, reporting of data on age and sex of patients with UGS is erratic at best, with several papers failing to report information on sex and age. If data were presented, summary metrics used were different between similar sized populations.

**Table 1:** Summary of the complications, number of studies regarding them and number of described patients

	Diagnosis		Treatment	
	Studies	Patients	Studies	Patients
Bladder cancer	45	4314	14	93
Ectopic pregnancy	13	17	15	21
Infertility	19	154	11	13
Invasive procedures	43	75	17	45
Kidney failure	2	48	5	35
Obstructive uropathy	31	3142	23	3415
Urolithiasis	3	464	2	26

**Table 2:** Summary of studies with at least 10 participants describing the diagnosis of cUGS patients, excluding studies on schistosomiasis-related bladder cancer which are reported in detail in Table 3. Age refers to the whole cohort studied in the article (not just the complicated patients) when in italic

Refs	Author	Year	Country	Study type	Population	Methodology	Sex	Age	Main findings	Graphical representation
Obstructive Uropathy (OU)										
[56]	Abdel-Wahab MF	1992	Egypt	Transversal study	Endemic area residents	Mass screening of children for the presence of OU by US	m/f	*Range: [12–16]*	Amongst 422 children with a history of schistosomiasis or a documented infection, UO was found in 22 subjects (5.2%), with prevalences ranging from 5% (uninfected, untreated group) to 17% (heavily infected). The study highlights a Potential role of US as a screening tool for OU in children with schistosomiasis	△
[37]	Abdel-Wahab MF	1992	Egypt	Transversal study	Endemic area residents	Ultrasound in the detection of OU in forty patients with various manifestations of cUGS. Schistosomiasis diagnosed by egg count in the urine	m/f	Mean [range]: 43.6 [15–66] (OU)	Amongst 40 patients with documented infection, sensitivity of 89% and a specificity of 64% in detecting OU compared to traditional radiology Practical role of US in the detection of OU and other findings related to chronic schistosomiasis	▵
[57]	Brouwer KC	2003	Zimbabwe	Transversal study	Endemic area residents	Genetic profiling of 73 miracidial isolates from patients with urinary damage vs 60 isolates from patients without urinary damage	m/f	*Range: [9–16]*	Patients with heavy infections harboured a higher number of strains. Three parasitic clusters were found to be more closely associated with pathology and the presence of UO	NA
[28]	Brouwer KC	2003	Zimbabwe	Transversal study	Endemic area residents	Ultrasound of 329 students with confirmed *S. haematobium* infection to detect the presence of OU in children in an hyperendemic area	m/f	*Range: [9–16]*	UO prevalence of 36% in infected children. Bladder pathology was found in 27% of the students. The study confirms the role of US in the detection of OU in children. The authors suggest US to be used to predict disease severity and for targeting treatment to those most at risk	△
[19]	Dabo A	1995	Mali	Transversal study	Endemic area residents	Ultrasound in 266 children to detect the presence of OU in children in an hyperendemic area	m/f	*Range: [6–15]*	59/266 (22.1%) of children had OU. The study showed a good performance at detecting OU in children and adolescents. Authors also found a direct correlation between intensity of infection assessed by egg count and degree of hydronephrosis	△
[48]	Fataar S	1990	Kuwait	Transversal study	Endemic area residents	CT scan in ten patients with confirmed infection to evaluate alterations visible in genitourinary schistosomiasis	m/f	Mean [range]: 30 [21–50]	All patients presented with calcifications. One patient had UO. CT can be used to evaluate the presence of OU and other findings related to chronic schistosomiasis	▵
[20]	Garba A	1999	Burkina Faso	Transversal study	Endemic area residents	Ultrasound on 203 children with confirmed infection to detect the presence of OU in children in an hyperendemic area	m/f	*Range: [7–15]*	The authors only report a prevalence of 2% of OU seen by US. Confirmation of potential role of US as a screening tool for OU	△
[36]	Ibrahim AI	1991	Saudi Arabia	Transversal study	Endemic area residents	Micturating cystourethrogram in 47 patients to study the presence of OU in chronic Schistosomiasis	m/f	Mean [range]: 34.6 [18–68]	Thirteen patients (27%) were found to have OU. Method is no longer of choice by current practices. The authors question the relevance of OU as a morbidity factor in cUGS	NA
[21]	Rasendramino MH	1998	Madagascar	Transversal study	Endemic area residents	Ultrasound on 436 infected patients to detect the presence of OU in residents in an hyperendemic area	m/f	Unspecified	5.3% of patients had a dilated ureter, 14% of patients had kidney OU. Practical role of US in the detection of OU and other findings related to chronic schistosomiasis	△
[22]	Remppis J	2020	Gabon	Transversal study	Endemic area residents	Development of an ultrasonographic protocol to assess schistosome-related urinary tract pathology, tested on an endemic population by a clinician with little US experience and a medical student with no US experience (images were reviewed by two experts),	m/f	Unspecified	A focussed assessment of bladder pathology in OU performed on 110 patients. Good feasibility in non-expert operator with around 20 h of training. UO detection showed sensitivity of 83 and 100% in trained operators, with a specificity of 98 and 100%. Similar values for ureteral OU when proximal pathology, whilst values dropped significantly for distal pathology (Se 17 and 23%)	△
[23]	Salah MA	2000	Yemen	Transversal study	Endemic area residents	Ultrasound to detect the presence of OU in residents in an endemic area 158 included patients	m/f	*Mean [range]: 17 [6–41]*	OU found in 12% of patients (upper urinary tract) and 16% of patients. Practical role of US in the detection of OU related to chronic schistosomiasis	△
[26]	Salas-Coronas J	2013	Spain	Retrospective study	Migrants	Ultrasound to detect the presence of OU in migrants residing in non- endemic area (219 patients)	m/f	*Mean [range]: 26.7 [15–52]*	The authors only report a prevalence of 1.1% of OU in migrants with schistosomiasis seen by US	△
[12]	Salas-Coronas J	2020	Spain	Retrospective study	Migrants	Ultrasound to detect the presence of OU in a non-endemic area (386 patients, partially overlapping with the previous cohort)	m/f	*Mean [range]: 27.3 [11–57]*	The authors only report a prevalence of 1.5% of OU in migrants with schistosomiasis seen by US	△
[24]	Serieye J	1996	Madagascar	Transversal study	Endemic area residents	Ultrasound to detect the presence of OU in residents in an hyperendemic area (574 patients, with a prevalence of S. haematobium infection of 75,9% of patients) and in a control are (100 patients, 7 of whom infected)	m/f	> 5	Good performance at detecting OU in adults (11.6% of patients with “congestive changes”), direct correlation between intensity of infection assessed by egg count and degree of hydronephrosis	△
[25]	Vester U	1997	Mali	Transversal study	Endemic area residents	Ultrasound to detect the presence of OU in residents in an untreated hyperendemic area in 776 patients with confirmed infection	m/f	> 2	OU found in 6 to 23% of patients, with a decreasing prevalence with older age. Pelvic dilation found in 4 to 15% of patients, frank hydronephrosis found in 1.3% of patients. Practical role of US in the detection of OU and other findings related to chronic schistosomiasis	△
Schistosome-related bladder cancer										
[37]	Abdel-Wahab MF	1992	Egypt	Transversal study	Endemic area residents	Ultrasound in the detection of OU in forty patients with various manifestations of cUGS. Ten patients had a bladder mass, nine of which in this case were cancers. Sensitivity of 89% and a specificity of 100% in detecting OU compared to traditional radiology	m/f	Mean [range]: 60.4 [49–80]	Practical role of US in the detection of bladder masses underlying cancers related to chronic schistosomiasis	▵
[158]	Ahmed NS	2017	Egypt	Retrospective study	Endemic area residents	Study carried out to analyze the histopathological changes in the urinary bladder affected by *Schistosoma haematobium*	m/f	*Range: [20–64]*	Biopsy revealed invasive squamous carcinoma in 11/54 people with histological changes	NA
[61]	Ahmed SA	1996	Egypt	Transversal study	Endemic area residents	Serum LDH and amino acid patterns evaluated in small cohorts of patients with schistosomiasis-associated cancers (*n* = 6), active schistosomiasis and no evidence of cancer (*n* = 22), non-schistosomal bladder cancer (*n* = 6) and uninfected patients with hepatocellular carcinoma (*n* = 13). Amongst the active schistosomiasis groups hepatic comorbidities were present in nine patients. Chronic schistosomiasis patients were also included	Unspecified	Unspecified	No significant difference in LDH levels amongst different pathologies, although it may be useful to monitor patient status. No amino acidic pattern differentiates chronic bilharziasis from schistosomal bladder cancer patients	◀▶
[59]	Akinwale OP	2008	Nigeria	Transversal study	Endemic area residents	Cytology to screen exfoliated cells in the urines of 32 infected individuals and 10 uninfected controls	m/f	*Mean [range]: 47.5 [40–74]*	Cytology can identify severely dysplastic to frankly malignant squamous cells, but has a low sensitivity especially for low grade and low stage ones (only 9.4% of patients had alterations)	◃▹
[159]	Al-Samawi AS	2013	Yemen	Transversal study	Endemic area residents	Study carried out to describe the clinicopathological features of bladder cancers diagnosed at a centre in Yemen	m/f	*Mean [range]: 57.6 [12–99]*	Thirty-one out of seventy-five squamous cell carcinomas of the bladder were described as showing histological evidence of schistosomal eggs in biopsies or surgical specimens	NA
[160]	Amin HAA	2019	Egypt	Transversal study	Endemic area residents	Observational histological study to investigate the characteristics of bladder cancer in Egypt	m/f	*Mean [range]: 61.6 [20–82]*	Nineteen out of 87 (21.9%). Bladder cancers were positive for histological evidence of schistosomal eggs in biopsies or surgical specimens, whilst 8 were histologically described as showing suggestive features for schistosomiasis	NA
[161]	Bedwani R	1998	Egypt	Case–control study	Endemic area residents	Eggs were searched in histological samples of patients with bladder cancer and in the urine of a control group to investigate the relationship between history of schistosomiasis and bladder cancer risk	m/f	*Median [range]: 59 [21–78]*	Eggs in histological samples were found in 86/190 (45%) bladder cancer patients	NA
[162]	Darré T	2015	Togo	Retrospective study	Endemic area residents	Description of the histological specimens coming from bladder lesions observed during a 10-year time span	Unspecified	Unspecified	Although most biopsies or surgical specimens were positive for eggs, 54 out of 192 (28.12%) bladder cancers were deemed to be caused by a chronic infection. The etiopathogenetic mechanism is not described for the other lesions	NA
[163]	Gaye AM	2016	Senegal	Retrospective study	Endemic area residents	Search of *S. haematobium* eggs in histological specimens of bladder cancers observed during a 5-year time span	Unspecified	Unspecified	31 of the 105 (29.5%) cases of bladder cancer were described as showing histological evidence of schistosomal eggs in biopsies or surgical specimens	NA
[164]	Groeneveld AE	1996	Burkina Faso	Retrospective study	Endemic area residents	Description of the histological specimens coming from bladder cancers observed during a 10-year time span	m/f	*Mean: 62.1*	197 of the 615 cases of bladder cancer (30.3%) were described as showing histological evidence of schistosomal eggs in biopsies	NA
[165]	Martin JW	2018	Egypt	Retrospective study	Endemic area residents	Description of the histological specimens coming from radical cystectomies due to bladder cancers observed during a 7-year time span	m/f	*Mean [range]: 54 [20–91]*	802 (64.8%) of the 1238 cases of bladder cancer were described as showing histological evidence of schistosomal eggs in surgical specimens	NA
[166]	Mungadi IA	2007	Nigeria	Retrospective study	Endemic area residents	Eggs were searched in histological samples of patients with bladder cancer to assess the impact of schistosomiasis on these cases	m/f	*Mean [range]: 46.0 [20–86]*	16 of the 43 (37.2%) cases of bladder cancer were described as showing histological evidence of schistosomal eggs in biopsies	NA
[58]	Santos J	2015	Angola	Transversal study	Endemic area residents	Cystoscopy to investigate eighty *S. haematobium*-infected patients with haematuria and bladder wall irregularities as seen by US. Eighty patients with confirmed *S. haematobium* infection	m/f	*Median [range]: 41 [3–79]*	The presence of one or more masses as seen by US examination correlated with a neoplasm with a sensitivity of 100% and a specificity of 73% Practical role of US in screening cases of haematuria due to schistosome-related bladder cancer	△
[62]	Yang H	2005	Egypt	Retrospective study	Endemic area residents	Human papillomavirus (HPV) DNA was searched in serum and urine of individual with schistosomal bladder cancer, cervical and head/neck cancers and controls. Twenty-seven patients with *Schistosoma*-associated bladder cancer and equal n of healthy controls and patients with *S. haematobium* infection	Unspecified	Unspecified	HPV DNA in serum had 96.3% sensitivity in detecting schistosomal bladder cancer, whilst HPV DNA in urine had a sensitivity of 62.5%. Both techniques had low specificity. It was noted that HPV DNA in serum could be used to monitor treatment of this disorder (no DNA was found in the sera of surgical patients within 2 weeks of surgical removal of a cancerous bladder)	▵
Female infertility										
[90]	Kjetland EF	1996	Malawi	Transversal study	Endemic area residents	Genital biopsies to confirm the presence of ova in the reproductive system of infertile women with eggs in the urine	f	*Median [range]: 22 [15–51]*	Most women with urinary schistosomiasis (egg in the urine) also had genital manifestations, independent of the intensity and degree of urinary disease. Urinary microscopy could be the first-line investigation for infertile women from endemic areas, once common STDs have been excluded	NA
[89]	Kjetland EF	2010	Zimbabwe	Transversal study	Endemic area residents	Cervical smear to detect the presence of *S. haematobium* eggs in infertile women from an endemic area. 434 patients, 15% with a diagnosis of infertility	f	*Range: [20–53]*	23/65 (35%) women were infertile Genital schistosomiasis in women is associated with infertility, but cervical smears have low-sensitivity for its diagnosis, on clinical indication a biopsy should be used	NA
[81]	Nayama M	2007	Niger	Transversal study	Endemic area residents	42 infertile women with schistosomiasis from an endemic area were surveyed for genital symptoms; 26 of them were biopsied and 11 underwent a hysterosalpingography	f	*Mean [range]: 26 [15–44]*	Clinically evident exocervicites are associated with genital schistosomiasis as it was found in 21 patients (50%). The infestation may lead to ovarian cysts or dystrophy and adhesions (visible at hysterosalpingography), which are the cause to schistosomiasis-related infertility	NA
[157]	Santos J	2014	Angola	Transversal study	Endemic area residents	Dosing oestrogen-like metabolites in urines of women with urinary schistosomiasis (*n* = 87) and in a control group (*n* = 87)	f	Mean [range]: 25.6 [18–45]	The catechol–oestrogens/ DNA adducts were significantly associated with schistosomiasis, as they were found in 17 (19.5%) vs 8 (9.2%) patients in the two groups. In addition, presence of these metabolites was positively associated with infertility	▵

Data are presented as summaries of findings using Effect Direction Plots [17]. Sample size: large arrow > 300; medium arrow 50–300; small arrow < 50. Effect direction: upward arrow = increased diagnostic capabilities considering the operational context, downward arrow = increased diagnostic capabilities considering the operational context, sideways arrow = no change/conflicting findings

**Table 3:** Studies regarding urinary markers for UGS-related bladder cancer. Age refers to the whole cohort studied in the article (not just the complicated patients) when in italic

Ref	Urinary marker	Cases vs controls, statistical significance y/n?	SBC vs NSBC, statistical significance	Sex	Age	n	Se	Sp	PPV	NPV
[167]	Nitrate	Yes	No	m	Mean ± SD: 54 ± 10	61	0.74	0.07	0.45	0.2
	Nitrite	Yes	No			61	1	0.33	0.61	1
	Apparent total N-nitroso compounds	Yes	No			61	0.97	0.07	0.52	0.67
	N-nitrosodimethylamine	Yes	Yes			61	1	0	0.51	0
	N-nitrosopiperidine	No	–			61	0.26	0.43	0.32	0.36
	N-nitrosopyrrolidine	No	–			61	0.26	0.5	0.35	0.39
	N-nitrosodibutylamine	No	–			61	0.26	0.6	0.4	0.44
[168]	Sha-miR-71a	Yes	Yes	m/f	Mean ± SD [range]: 61 ± 10 [44–79]	50	0.89	1	1	0.79
	MAPK3	Yes	Yes			85	0.86	1	1	0.91
	MAPK3 mRNA	Yes	Yes			85				
[60]	CEA	Yes	Yes	Unspecified	Unspecified	32	0.86	1	1	0.77
[169]	BTA stat	Yes	No	Unspecified	*Mean [range]: 57 [26–89]*	85	0.98	0.15	0.79	0.75
	BTA TRAK	Yes	No			85	0.94	0.15	0.78	0.43
[170]	MMP-3	Yes	No	m/f	Mean [range]: 62.8 [38–88]	82				
	MMP-9	Yes	No			82				
[171]	MMP-2	Yes	No	m/f	*Mean* ± *SD: 54* ± *8*	166				
	MMP-9	Yes	No			166				
	MMP-9/NGAL	Yes	No			166				
	MMP-9 dimers	Yes	No			166				
	MMP-9/TIMP-1	No	–			166				
	ADAMTS-7	No	–			166				
[172]	MMP-2	Yes	No	m/f	*Mean* ± *SD [range]: 57* ± *12 [25–86]*	224				
	MMP-9	Yes	No			224				
	MMP-2/TIMP-2	Yes	Yes			224				
	MMP-9/TIMP-2	Yes	No			224				
[173]	Telomerase activity	Yes	Yes	Unspecified	*Mean [range]: 58 [30–52]*	100				
	MMP-9	Yes	Yes			95				
[174]	NMP22	Yes	Yes	Unspecified	*Mean [range]: 56.5 [30–80]*	215				
	Fibronectin	Yes	Yes			215				
	UBC	Yes	Yes			215				
[175]	Telomerase activity	Yes	Yes	m/f	*Mean* ± *SD [range]: 57.8* ± *10 [36–82]*	243	0.76	0.76	0.86	0.63
	Telomerase mRNA	Yes	Yes			243	0.94	0.88	0.94	0.88
	hTERT mRNA	Yes	Yes			243	0.97	0.88	0.94	0.95
[176]	hTERT mRNA	Yes	No	m/f	*Mean* ± *SD [range]: 58.1* ± *10.2 [42–82]*	105				
	SF	Yes	No			105				
[177]	lncRNA-UCA1	Yes	No	m/f	*Mean* ± *SD [range]: 61.8* ± *8.2 [40–84]*	56	1	1	1	1
[178]	miRNA-210	Yes	No	Unspecified	Unspecified	58	0.79	1	1	0.71
	miRNA-96	Yes	No			58	0.68	1	1	0.63
	lncRNA-UCA1	Yes	No			58	1	1	1	1
	Hyaluronidase mRNA	Yes	No			58	0.84	1	1	0.77
[179]	miRNA-96	Yes	No	m/f	*Mean* ± *SD [range]: 58.8* ± *11.6 [28–89]*	47	0.77	0.67	0.87	0.5
[180]	miRNA-210	Yes	No	Unspecified	Unspecified	96	0.64	0.83	0.92	0.43
	miRNA-10b	Yes	No			96	0.78	1	1	0.6
	miRNA-29c	Yes	Yes			96	0.81	1	1	0.63
[181]	Hyaluronidase mRNA	Yes	Yes	m/f	*Mean* ± *SD [range]: 58.0* ± *10.2 [35–86]*	274	0.92	0.9	0.96	0.79
	CK-20 mRNA	Yes	No			274	0.79	0.97	0.99	0.6
[182]	Survivin	Yes	No	m/f	*Mean* ± *SD [range]: 60.1* ± *11.8 [26–87]*	100				
	Hyaluronidase mRNA	Yes	No			100				
[183]	CK-20 mRNA	Yes	No	mf	*Mean* ± *SD [range]: 55.9* ± *8.8 [40–82]*	131				
	Angiogenin	Yes	No			131				
[184]	CD44	Yes	Yes	Unspecified	*Mean* ± *SD [range]: 56.6* ± *9.6 [36–84]*	156				
	CK-20 mRNA	Yes	No			156				
[185]	HURP mRNA	Yes	No	m/f	*Mean* ± *SD [range]: 52* ± *10 [25–83]*	151	0.74	0.91	0.98	0.38
[186]	ATG12	Yes	No	m/f	Unspecified	66	0.81	1	1	0.58
	FYCO1	Yes	No			66	0.85	0.57	0.88	0.5
	ULK1	Yes	Yes			66	0.92	1	1	0.78
	CT scanPR	Yes	Yes			66	0.96	1	1	0.88
[187]	CYFRA21-1	No	–	m/f	*Range: [35–82]*	270	0.83	0.58	0.56	0.84

Values refer to the ability of the tests to distinguish a schistosomal bladder cancer in a sample consisting of schistosomal bladder cancers, non-schistosomal bladder cancers, schistosomiasis without cancers and healthy controls

## Diagnosis

### Obstructive uropathy, kidney failure and urolithiasis

Ultrasound (US) was the most commonly used to diagnose obstructive uropathy (OU). US was employed following the Niamey protocol [18] in 19 studies over the 31 found to deal with the diagnosis of OU. Other radiological methods found to be used were CT scans (8 studies), intravenous urography (7 studies), renal scintigraphy (2 studies) and cystography (2 studies). Plain X-rays were used in 8 reports. Whilst 12 studies have used US alone for the diagnosis of OU [12, 19–29], others have used two methods to confirm the complication (intravenous urography in five case reports [30–34], anterior urography in one patient from a case series [33], CT scan in one case report [35]) or an invasive procedure (cystoscopy in one case report [35]). Very few studies compared the performances of radiological methods: only one study compared the performances of intravenous urography (considered the gold standard) and cystography, finding suboptimal performances of the latter (Sensitivity (Se) 26.8%; specificity (Sp) 66.7%; positive predictive value (PPV) 84.6%; negative predictive value (NPV) 11.8%) [36]. US proved superior to intravenous urography as one study found better performances (Se 89.5%; Sp 63.6%; PPV 81.0%; NPV 77.8%) and the ability to show hydronephrosis in patients whose kidneys were not visible via intravenous urography [37]. As said, intravenous urography has mainly been used as a gold standard in evaluating other diagnostic techniques [36, 37] or to confirm ultrasonographic results [30–34]. One study assessed the sensitivity and specificity of cystography for the diagnosis of kidney failure in the setting of OU, but found no statistical difference in renal deterioration between patients with and without vesicoureteral reflux [36]. A case report [38] illustrated a case of *S. haematobium*-associated glomerulopathy, a phenomenon more frequently associated with *S. mansoni* [39–47]. One study on 10 patients looked at the ability of CT scans to show the typical alterations of UGS, including OU [48]. CT scans were also described with comparable results in another 8 case reports [35, 49–55].

Some studies tried to use non-radiological means to predict the presence of OU: one study assessed the role of egg burden (proposing a cut-off of 10 eggs/ml of urine). However, the use of this marker showed a very low sensitivity (25%) when compared to US with an expected good specificity (93%) [56]. Another study tried to find a correlation between the genetic composition of the parasitic population and the presence of hydronephrosis, with the presence of some genetic clusters showing a sensitivity of 81.8% and the absence of cluster 7 showing a sensitivity of 90.9% [57].

Stones in the urinary tract were reported as cUGS in three articles [26, 37, 56]. Two reports compared US to plain X-rays to diagnose the presence of stones, with a sensitivity of 66.7 and 70%, respectively, and better specificity (90 and 97%) [26, 37]. As expected, stones in the ureter were less easy to spot by US whilst a sensitivity of 100% was found in searching for stones in the bladder in one study [37]. Supplementary Table 1 shows all the papers dealing with the diagnosis of OU. Visual effect plots for studies with at least 10 participants dealing with the diagnosis of UO are presented in Table 2.

### Bladder cancer

Forty-five studies dealt with the diagnosis of bladder cancer. The most commonly employed radiological method was US. All articles reported on the presence of cancers associated to schistosomiasis confirmed by histological examination. Thirty-six studies also reported data on the use of other techniques. Radiological methods included US in five studies, CT scan in six studies, positron emission tomography (PET) in one case report and urography in one case report. Twenty-two studies evaluated the role of urinary markers, whilst one study only relied on urinary cytology. Cystoscopy was used in three studies. Again, studies comparing different methods were scarce, with one study comparing US to cystoscopy on eighty patients with confirmed squamous cell carcinomas and *S. haematobium* infection showed a good sensitivity (88.9%) and excellent specificity (100%) in detecting masses seen at cystoscopy [58]. The only study evaluating the use of urinary cytology detected alterations in 3 of 32 patients with bladder cancer and schistosomiasis [59]. Supplementary Table 2 shows all the papers dealing with the diagnosis of urinary cancers associated with schistosomiasis.

Amongst studies evaluating non-instrumental tools for the diagnosis of *Schistosoma*-related bladder cancer, one report evaluated the dosage of urinary CEA. Eighty-six per cent of patients with *Schistosoma*-related bladder cancer presented elevated value of urinary CEA, compared to 62% of patient with bladder cancer not related to *Schistosoma* and 0% of controls without cancer nor schistosomiasis [60]. Although the authors did not test for differences between schistosomal and non-*Schistosoma*-associated bladder cancers, there was no statistical difference applying a *t* test to the data presented in the article (*p* = 0.1076, mean CEA 74.27 ± 64.96 SD vs 43.86 ± 55.66 SD). LDH was employed in one study, but no differences were found comparing patients with schistosomiasis-associated cancers with patients with active schistosomiasis and no evidence of cancer, patients with non-*Schistosoma* associated bladder cancer and uninfected patients with hepatocellular carcinoma [61]. Several studies (Table 3) explored the use of a plethora of urinary markers to distinguish cancers due to chronic schistosomiasis and those due to other causes. However, all these studies were limited by the lack of data on the presence of other risk factors for cancer. A small pilot study (6 patients per group) on differences in the circulating amino acids pattern in patients with schistosomal squamocellular bladder cancer, chronic UGS patients and controls found that there were differences between UGS patients and controls, but no differences between chronic UGS and cancer patients [61]. Another study found an increase in the levels of HPV-17 DNA in the serum of UGS cancer patients compared to both healthy controls and non-UGS cancer patients. However, numbers in this study were also small with 24 patients per group [62]. Excluding studies on schistosomiasis-related bladder cancer which are reported in details in Table 3, visual effect plots for additional eligible studies with at least 10 participants dealing with the diagnosis of bladder cancers are presented in Table 2**.**

### Ectopic pregnancy and infertility

Data on diagnosis of ectopic pregnancy were reported in 13 articles, all case reports or case series. In all instances, the presence of *Schistosoma* eggs was detected only after surgery, when histology was performed. Diagnoses were made dosing β-HCG levels, clinically or at US [63–75]. Of note, no women were screened for the presence of *Schistosoma* eggs in urine before the procedure, and only in two instances, eggs were searched for after the intervention: in one case (a migrant woman recently immigrated to the United States from Africa), eggs were present in urine [63], and in another, patient (a traveller) urine samples were negative [68].

Male infertility was the subject of two case reports. In one instance, the correlation with *Schistosoma* infection was made due to the presence of granulomas seen at histological examination of a testicular mass [76]. In the other, azoospermia was deemed obstructive after testosterone and gonadotropin dosing and the causal relationship was inferred due to the history of schistosomiasis. This single report does not mention results from serology or parasitological results [77].

Female infertility was the subject of 17 articles, mostly case reports (*n* = 7) or case series (*n* = 3). Various techniques were used to characterize the diagnosis of infertility, including US (both transabdominal [78] and transvaginal [79, 80], CT scan, [80] hysterosalpingography [79, 81–84] and hysteroscopy [65]. In the ten case reports/series, the diagnosis was made after histological examination of either biopsies or surgical specimens of the fallopian tubes [65, 79, 80, 83–87], ovaries [79, 82, 84–86], peritoneum [65, 85], and cervix [88]. Reasons for these invasive procedures included ultrasonographic or radiographic evidence of hydrosalpinges and subsequent surgery [65, 79, 80, 83–85] and an atypical laparoscopic aspect of a lesion thought to be endometriosis [86]. In five patients, egg search in the urine was performed, but was negative in all instances [65, 85, 86].

In one cross-sectional study of 109 women with infertility, a clinical diagnosis was employed considering a combination of history of haematuria, genital or urological lesions detected by US and a positive eggs count in urine to identify cases, with 13 women having a positive eggs count over the whole cohort of 109 patients (11.9%). All the 42 cervical smears in this study were negative for *S. haematobium* eggs, whilst two biopsies were positive for the presence of eggs [81]. Another study found that amongst 23 women with infertility, 4 had a smear positive for eggs presence [89]. The latter study concluded that there was a significant association between the presence of eggs and infertility. In another study using histological positivity for *S. haematobium* eggs in cervical biopsies as the definition for female genital schistosomiasis, 19 of 31 women were found to be infertile. Five women with negative cervical biopsies and eggs in urine were also infertile [90]. Supplementary Table 3 shows a summary of all articles dealing with ectopic pregnancy and male or female infertility. No papers dealing with abortions were retrieved. Visual effect plots for eligible articles dealing with the diagnosis of female infertility are presented in Table 2.

### Invasive procedures

Twenty-four patients underwent invasive procedures in the suspect of a disease different than schistosomiasis (e.g. bladder cancer unrelated to schistosomiasis). Most patients (20 of 24, 86%) were migrants from endemic countries. Most cases (19 of 24, 70%) were examined after urogenital manifestations seen clinically or by US (*n* = 9 granulomatous lesions observed at US examination, *n* = 5 polyps observed at US, *n* = 4 masses observed at US, *n* = 1 persistent haematuria). Serology was performed only in two patients and was positive in both [91, 92]. Urine filtration and eggs search was performed in 15 patients and was positive in nine (60%) [92–106]. A summary of this studies is presented in Supplementary Table 4.

## Treatment

### Obstructive uropathy, urolithiasis and kidney failure

Twenty-three articles concerned the treatment of OU in cUGS patients. Two articles (8.7%) reported the use of medical anthelmintic treatment, with no distinction made over the use of PZQ or metrifonate, eight (34.3%) only reported using PZQ and one (4.3%) only metrifonate. Twelve articles (47.8%) dealt with the use of surgery, one using robotic surgery. Laser endoureterotomy followed by stenting was used in five studies (23%).

Studies on medical therapy showed varying methodologies. Some studies did not allow the use of individual-patient data as authors evaluated the effects of mass drug administration (MDA) on the prevalence of OU by US. One study conducted in Madagascar using PZQ (*n* = 472) showed the prevalence of OU reducing from 13.6 to 2.6% twelve months after the MDA campaign [107]. Another study in Niger staged an MDA intervention in adolescents and adults of two villages with a total population of 2570 people, 72.3% were treated using a single dose of 40 mg/kg PZQ. This led to a reduction of OU prevalence from 17 to 4% 36 months after treatment [108]. Studies with longer follow-up periods showed contrasting results. In a study involving a cohort of patients aged 14–19 and remotely treated with a single dose of PZQ or metrifonate in the context of an RCT followed-up for ten years (*n* = 132), the prevalence (0.7%) of hydronephrosis was the same as the untreated cohort [109]. Another study followed a cohort of 194 patients for thirteen years after receiving either PZQ or metrifonate. In this study, prevalence dropped to zero, starting from 14% [110].

One study analyzed the efficacy of the Boari flap technique in ureteral re-implantation surgeries for patients with chronic stenosis. In this study, 2 out of 150 patients (1.3%) had complications (one urinoma, one sepsis post-surgery) [111]. Nephrectomy was reported for two patients, but no surgical outcomes are presented in the two articles [13, 50]. In two patients, a bladder reconstruction with the creation of an ostomy, with the patient improving in the long term [31, 53].

Concerning the use of endoscopic techniques, one randomized study compared the use of two ipsilateral double-J stents vs a single double-J stent after laser endoureterotomy for the treatment of ureteral stenosis due to schistosomiasis. The use of two stents improved outcomes and was found to be most useful when the stenotic tract was longer than 1.5 cm [112].

A total of eleven patients treated with combination therapy were present in 9 case reports or series. In seven cases, a combined treatment of surgical and medical therapy was employed [30, 32–34, 49–51], the other patients were only treated surgically [35, 113]. Surgical techniques included all those found in prospective and retrospective studies (i.e. ureteral re-implantation or reconstruction or nephrectomy), and only two patients had a worsening of OU after treatment or a complication [33, 49].

Two articles concerned the treatment of urolithiasis in cUGS. One case series described the stone removal by surgical lithotomy in 17 patients, whilst two required ureteral re-implantation and two had to undergo a nephrectomy. All 21 patients improved after treatment, despite 3 developing surgical-site infections [114]. One article illustrated the use of percutaneous suprapubic cystolithotripsy as the treatment of choice for bladder stones secondary to schistosomiasis in a prospective cohort of 5 paediatric patients; all of them fully recovered without complications [115].

Four articles concerned the treatment of kidney failure. In one report on a patient with OU and AKI due to hydronephrosis, dialysis was employed followed by a percutaneous ostomy to treat the patient [54]. In another report, CKD led to the patient starting dialysis and subsequently died of sepsis [12].

A single case–control study, intended to examine the safety of three monthly doses of 60 mg/kg PZQ and their efficacy in reducing the eggs count, also examined the effect of this therapy on kidney function of 28 *S. haematobium*-infected patients with no renal damage. The study showed improvements of post-treatment organ function (increase of the eGFR) without significant adverse effects [116].

In a case series of four patients with CKD due to OU, two patients were managed by ureter replacement by ureteroileoplasty, whilst in two cases nephrectomy was employed. All four patients then went on to require transplantation [117].

One case of nephrotic syndrome due to *S. haematobium* was found. The patient was managed using cyclophosphamide, methylprednisone, and PZQ but still reached end-stage disease and required transplantation [38]. Of note 9 articles reporting data on glomerular disease were excluded because the etiological agent was *S. mansoni* [39–47]. Supplementary Table 5 shows a summary of all articles regarding the treatment of UO. Visual effect plots for eligible articles dealing with the treatment of UO are presented in Table 4.

**Table 4:** Summary of studies with at least 10 participants describing the therapy of cUGS patients. Age refers to the whole cohort studied in the article (not just the complicated patients) when in italic

Refs	Author	Year	Country	Study type	Population	Methodology	Sex	Age	Main findings	Graphical representation
Obstructive Uropathy (OU)										
[108]	Garba A	2004	Niger	Prospective cohort	Endemic area residents	MDA with Praziquantel (PZQ) and its effects on the prevalence of OU in people of different ages. Total n of patients 872, but it is impossible to know if the same patient was treated twice. Prevalence of *S. haematobium* infection ranging from 30.4% to 74.1% depending on the year	Unspecified	Unspecified	Decrease of the prevalence of OU from a global 17% to a global 4% during MDA campaign. In children ≤ 15 years old, prevalence reduced from 22.0% to 4.4%; in people > 15 y.o. it reduced from 12.3% to 3.5%. It is however impossible to estimate single patient effects due to the nature of the study	△
[142]	King CH	2002	Kenya	RCT	Endemic area residents	PZQ at two different doses (20 mg/kg SD vs 40 mg/kg SD) in the treatment of OU in 23 patients	m/f	*Range: [4–23]*	Small study (9 vs 14 patients) with equal efficacy for the two regimens in reducing OU. Authors conclude that a 20 mg/kg dose may be sufficient in providing control of urinary morbidity	▴
[112]	Mohyelden K	2020	Egypt	RCT	Endemic area residents	Comparison between a single Double-J stent and two ipsilateral Double-J stents after laser endo-ureterotomy on three treatment of OU. 32 vs 31 patients	m/f	Mean ± SD: 43.6 ± 13.0	Insertion of two Double-J stents provides better long-term patency rates than insertion of a single Double-J stent, in patients with strictures longer than 1.5 cm (85.7% success vs 38.5%)	▲
[109]	Ouma JH	2005	Kenya	Case–control study	Endemic area residents	Comparison between 132 adults treated with either PZQ or metrifonate 10–18 years prior to the study and a control group (n = 132)	m/f	*Mean* ± *SD: 29.1* ± *7.5 (age at follow-up)*	Treated communities reported a prevalence of advanced kidney abnormalities by ultrasound of 0.7% compared to 0.7% in the untreated communities. Repeated therapy into adulthood may be required to fully control hydronephrosis in high-risk adult populations	◀▶
[107]	Rasendramino MH	1998	Madagascar	Prospective cohort	Endemic area residents	MDA with PZQ 40 mg/kg SD and its effects on the prevalence of OU (n = 453)	m/f	> 5	Reduction in the prevalence of OU (from 13.6% to 2.5%) 12 months after the MDA campaign. It is however impossible to estimate single patient effects due to the nature of the study	▲
[111]	Ravi G	1993	Saudi Arabia	Prospective cohort	Endemic area residents	One hundred and fifty patients treated with ureteric re-implantation using a Boari flap technique	m/f	Mean [range]: 35 [4–84]	The Boari flap technique had a success rate of 97.9% for the treatment of ureteric dilation, and 99.3% of patients had their symptoms disappeared or improved	△
[110]	Subramanian AK	1999	Kenya	Prospective cohort	Endemic area residents	Comparison between findings before the use of MDA with PZQ or metrifonate on 517 individuals (in 1984) and its effects on the prevalence of OU in the follow-up group (n = 194) seen in 1994	m/f	*median [range]: 24 [16–39] (age at follow-up)*	Hydronephrosis decreases from 17 to 7%, with no severe hydronephrosis cases persisting after treatment Treatment also reduced urinary tract morbidity despite re-infection. Authors suggest that reducing the duration of infection during early adolescence might protect against urinary tract morbidity	▲
Schistosome-related bladder cancer										
[188]	Abdou A	2012	France	Retrospective cohort	Travellers and migrants	Retrospective cohort of 241 patients with *S. haematobium*-related bladder cancers with various histology and at various stages of the disease, managed with cystectomies or palliative treatments	m/f	Mean [range]: 50 [37–69]	Mixed results not traceable back to a specific combination of histology-stage-treatment. Management of these cancers did not differ from those unrelated to schistosomiasis	NA
[189]	Aly MS	2012	France	Prospective cohort	Endemic area residents	41 patients with *Schistosoma*-associated bladder cancer in advanced and metastatic stages treated with gemcitabine-cisplatin observing their response related to chromosomal abnormalities	m/f	Mean ± SD; median [range]: 53.9 ± 9.5; 54.5 [39–81]	Mean time to progression was 7.82 months (median: 5 months); mean overall survival was 13.36 months (median: 10 months). Gain of chromosome 4 was the only chromosomal abnormality with a statistically significant reduction in overall survival	NA
[122]	Wishahi MM	1994	Egypt	Cohort study	Endemic area residents	Transurethral resection of the bladder followed by immunotherapy with BCG in 17 SCC patients with transitional cell carcinoma (Ta, T1 and Tis) and a control group (n = 17) with similar characteristics	m/f	Mean ± SD; median [range]: 42.1 ± 10.0; 43 [27–63]	Estimated decrease in the recurrence of tumours after intravesical BCG therapy (2 vs 1.65 recurrences/100 patients/month). The tumour-free period after BCG treatment was 15.7 months compared with 6.7 months in the control group	▴
[121]	Wishahi MM	1995	Egypt	Prospective cohort	Endemic area residents	Transurethral resection of the bladder followed by immunotherapy with keyhole limpet haemocyanin (KLH) in patients with transitional cell carcinoma (Ta, T1 and Tis)	m/f	Mean ± SD; median [range]: 45.0 ± 13.9; 39 [23–74]	KLH immunotherapy reduced the recurrence rate of superficial bladder tumours to 15.4% compared to 76.9% before therapy	▴
Schistosomiasis unresponsive to praziquantel (requiring biopsy)										
[135]	Silva IM	2005	Brazil		Travellers	Evaluation of an increased dose of PZQ after a first treatment at a 40 mg/kg dose. Treatment success verified by cystoscopy and subsequent histological visualization of granulomas and “viable” eggs	m/f	Median [range]: 29 [26–59]	26 patients treated with a single dose of 40 mg/kg PZQ underwent cystoscopy. 17 of them were cured, whilst the other 9 still had granulomas with vital eggs and were retreated with three doses of 40 mg/kg PZQ 15 days apart. Four of these nine patients had normal cystoscopies at a later evaluation. The authors postulate that PZQ efficacy increases proportionally to the administered dose: in areas with low efficacy of PZQ, the doses could be increased to three administration of 40 mg/kg	▵

Data are presented as summaries of findings using Effect Direction Plots [17]. Sample size: large arrow > 300; medium arrow 50–300; small arrow < 50. Effect direction: upward arrow = positive health impact, downward arrow = negative health impact, sideways arrow = no change/conflicting findings. Statistical significance: black arrow *p* < 0.05; grey arrow *p* > 0.05; empty arrow = no statistics/data reported. Age refers to the whole cohort studied in the article (not just the complicated patients) when in italic, since no patient-specific data were available

### Cancer

Reports on the treatment of schistosomiasis-related cancers were scarce. Only fourteen articles dealt with the treatment of cancer in cUGS patients, of which ten were case reports. Surgery was the most frequently employed treatment, followed by immunotherapy with Bacillus Calmette–Guérin (BCG, 1 article) or haemocyanin (1 article), an immunomodulator used to treat other urothelial tumours. Three case reports illustrated the use of combination therapy, particularly the use of chemotherapy followed by surgery and radiotherapy with lack of disease evidence at 19 months post-operation [118], trans-urethral resection of bladder (TURB) plus intra-vesical chemotherapy for a histologically mixed carcinoma [119] and finally surgery and adjuvant chemotherapy for a urothelial carcinoma, although outcome and follow-up information were not provided [120].

Two studies on immunomodulators showed promising results for both BCG (reduction of tumour recurrence, prolongation of the disease-free period by a median time of nine months) and haemocyanin, with a reduction of the relapse rate by 60% compared to endoscopic procedures not followed by immunotherapy [121, 122].

Supplementary Table 6 shows a summary of articles dealing with the treatment of bladder cancer associated with schistosomiasis. Visual effect plots for eligible articles dealing with the treatment of cancers associated with schistosomiasis are presented in Table 4.

### Abortion, ectopic pregnancy and infertility

No papers dealing with abortions were retrieved. Twenty-one patients had ectopic pregnancies linked to *S. haematobium* infections in 15 articles, all consisting of case reports or case series [63–69, 71–75, 84, 123, 124]. All cases were managed by salpingectomy with the employment of anthelmintics in 10 patients (47.6%, 9 patients treated with PZQ, 1 with niridazole) [63, 65, 67–69, 71–74, 123].

Both laparotomy [64, 67, 71–75, 84, 123] and laparoscopy [63, 65, 66, 68, 69, 75] were employed depending on settings and resources, as well as on the degree of urgency. In one case report, methotrexate was used given the increase of b-HCG post-surgery [68].

Concerning female infertility, 11 articles were found, all case reports describing a single patient. Surgical procedures of varying nature were employed (salpingectomies, neosalpingectomies, adhesiolysis and cyst excisions) [79, 80, 82–85]. For none of these therapies, fertility outcomes were reported. In two cases, besides surgical therapy, intra-cytoplasmic in vitro injection (ICSI) was also employed to attempt a fecundation. The procedure did not work in one case and its outcome was not reported in the other [80, 84].

Intracytoplasmic Sperm Injection (ICSI) was also employed in one of the two case reports on male infertility, where it allowed management of obstructive azoospermia linked to *S. haematobium* infection in a young male [77]. In another male patient, orchiectomy and PZQ were used to manage a granulomatous orchitis found after a testicular mass had been misdiagnosed as neoplastic. Sperm count and motility index, initially altered, returned to normality after treatment [76]. Supplementary Table 7 shows a summary of articles on the treatment of male and female genital complications associated with schistosomiasis.

### Patients diagnosed after invasive procedures

All the articles fitting this inclusion criteria were case reports or case series [85, 125–140] (Supplementary Table 8). One study [135] (Table 4) dealt with patient whose cUGS diagnosis was made after failure of one 40 mg/kg PZQ dose and a bladder biopsy showed the presence of granulomas with viable eggs. The authors argue that this points towards the adoption of a repeated dosing scheme in areas where PZQ resistance is known, but the study has limitations connected to the small number of patients included.

## Discussion

This review shows that the approaches for the diagnosis and management of cUGS are extremely variable, reflecting the protean clinical entity of the disease, able to affect different organs with different degrees of severity and requiring a case-by-case multidisciplinary approach in most patients.

Systematic data collection efforts are lacking: whilst we focussed on cUGS, the lack of standardization in the treatment of uncomplicated schistosomiasis and the lack of data gathering on treatment efficacy is also present [141]. Shared recommendations on the use of PZQ are for instance needed and should be widely adopted to increase the amount of usable data, with particular regard to their use in non-endemic settings where several different regimens are used (single or repeated doses with different timing) without solid efficacy data in support. [141]. Furthermore, in our review, most papers were either case reports or case series. Only two small RCTs were found [112, 142]. Absent outcome reporting is also problematic, as information is often lacking or incomplete (39 of 87 articles did not report follow-up data). When reported, its length rarely exceeded twelve months post intervention, which complicates efficacy estimates. Even basic demographic information was often lacking. Whilst a formal analysis of data on the participant’s age is impossible due to data heterogeneity, studies on OU diagnosis and treatment focus on younger subjects. Our findings on OU diagnosis and treatment might be less applicable to older patients. The same distinction emerges when we look at studies conducted in endemic countries versus studies in travellers, the former group being younger. This distinction is important as patients with a chronic infection are known to have poorer response to treatments in parasitic diseases [143, 144].

Exams found to be used for the diagnosis of cUGS in this review are characterized by low costs in most instances, as in the case of US for the visualization of the urinary tract [12, 19–27, 30–33, 35, 37]. US allows for a first level screening of OU and *Schistosoma*-related bladder cancers, with good sensitivities according to available data. US is also a diagnostic tool fitting the needs of resource-limited settings due to being cheap, repeatable and since it requires little logistics. Some authors have described the use of endo-venous uretherography for the diagnosis of OU. In this review, the technique was used as a gold standard to evaluate US as a diagnostic tool [36, 37], or alone in studies without comparator [30–34]. CT scans are also employed for the study of OU and other complications of the urinary tract [35, 48, 50–54]: their widespread application in endemic areas is hampered by costs and logistical issues. US has also been employed for the follow-up of patients with good performances in detecting disease regression for OU [108–110, 142, 145–147]. The same is true for bladder lesions, as reported by some authors [93, 148]. Altogether, available data support the use of US as an initial tool for the screening of cUGS including OU and bladder lesions. Pooled sensitivity data above 85% suggest that US could replace cystoscopy as a first-line exam in patients with a diagnosis of schistosomiasis, differently from previously stated in European guidelines [14].

The review also highlights that, even though late diagnosis and mismanagement were not the focus of this search, in several cases, the diagnosis of schistosomiasis was made only through histology whilst the most sensitive diagnostic test for the infection (which is serology) was often overlooked or performed after the diagnosis was already achieved.

Serology is used erratically in the diagnosis of patients with cUGS-related manifestations. In several reports [*n* = 7], patients underwent invasive procedures and were also found to have a positive serology for *S. haematobium* [85, 91, 92, 105, 137, 139, 149]. On the contrary, in several reports [*n* = 11], eggs were not present in the urine of patients with chronic schistosomiasis [92, 98, 100, 102, 103, 106, 126, 134, 137, 139, 140]. This is a known problem, as many of the pathologic manifestations in cUGS depend from chronic inflammation due to the formation of granulomas around eggs, rather than the continuous secretion of eggs from live parasites. No study found in this review employed the newest tool used in the diagnosis of *Schistosoma* infections, the circulating anodic antigen (CAA) assay [150]. This test has suggested to provide clinicians information on the presence of live adult parasites rather than just the presence of eggs, and its use could help guide treatment in patients with chronic schistosomiasis. Altogether, the results suggest the use of serology as a systematic tool for cUGS diagnosis, before the use of invasive procedures, whilst egg count in urine or other samples has shown low sensitivity for the diagnosis of cUGS. It is known that serology is the most sensible tool for the diagnosis of schistosomiasis [151] and may be employed as a cost-effective screening test in migrants recently arrived from highly endemic countries in order to identify those infected and treat them preventing the evolution to more advanced complicated disease [152]. The results of this review together with a recently published European multicenter case series showed that serology is positive in the majority of patients with cUGS confirming that this tool is also useful for the diagnosis of advanced disease [16]. Recently, molecular techniques have been increasingly used to diagnosed schistosomiasis; however, we did not find studies on cUGS where PCR was used, suggesting the need to study the role of molecular biology techniques in this cohort of patients.

A vast number of markers were used to diagnose *S. haematobium*-related cancers. A large part of the studies were carried out by the same group of authors from Egypt. Employed markers range from chemical compounds found to be altered in other pathological conditions (e.g. nitrates) to miRNAs (Table 3). External validation of these markers is required before they gain a place in clinical practice.

Several studies were carried out during elimination programmes, and PZQ was the most frequently employed drug to treat cUGS. Throughout this review, it was noted that the drug was employed at varying dosages, from the 40 mg/kg single dose recommended by the WHO to repeated courses used to treat more severe manifestations. Whilst data on the use of PZQ is lacking for genital cUGS, data on OU mostly coming from studies in the context of elimination programmes supports its use. A more systematic approach on data collection is needed to compare the overall efficacy of different regimes and dosage and effects of treatment on poorly explored outcome such as fertility.

This is even more important considering that available data suggests that the use of PZQ can revert some of the manifestations of cUGS, as shown by response rates for OU found in this review. Moreover, studies, especially case reports, did not report data on the follow-up of patients in 21.7% of articles [12, 13, 50, 53, 113]. 9.8% of patients with OU (333/3415) did not have data on follow-up, either because it is not mentioned in the article or because they did not show up to follow-up visits.

Concerning the effect of PZQ on OU, we found 8 eligible studies, 7 from endemic areas and 1 from Spain [33, 107, 108, 142, 145–147]. The largest study was carried out in Niger on 2570 subjects, about half under 15 years and half over 15 years of age. The study reveals that 3 years after a single treatment with PZQ 40 mg/kg, the prevalence of OU significantly decreases from 22 to 4.5% in children and from 12.3 to 3.6% in adults [108]. Another study carried out in Madagascar on 547 subjects reveals similar results decrease on prevalence of OU 12 months after treatment [107]. The remaining 6 studies involved a much smaller number of subjects, but confirmed that OU resolution may be observed 6–12 months after the treatment with PZQ [33, 142, 145–147]. However, several studies not matching our inclusion criteria suggest a positive effect of PZQ in patients whose OU grade was not definable [153–156].

The only trial found in this review compared two ipsilateral double-J stents and a single double-J stent for OU in schistosomiasis (in both cases stenting was performed after laser endoureterotomy). The first technique was found to be superior, concordantly with results on the treatment of OU causes by other etiologies (94). Despite the low number of patients considered, this trial may suggest that the management of patients with cUGS can be informed by results on complications caused by other etiologies.

In the few studies on infertile patients, screening was not done with serology, the most sensitive diagnostic method widely available to date [65, 70, 76–84, 86–90, 128, 157]. Most cases were diagnosed after invasive procedures. This also explains why only one case report described the use of PZQ as treatment for *Schistosoma-*related infertility [76].

This review has several limitations: we could not carry out a meta-analysis of collected data due to the nature of included studies. We included case reports to gather data on some of the most neglected manifestations of cUGS (e.g. infertility and ectopic pregnancy).

Overall, this survey shows an urgent need for prospective studies on several aspects of cUGS and that patients often suffer from a condition preventable by employing appropriate screening protocols for patients arriving in endemic areas (both migrants and returning travellers) as well as public health programmes in endemic areas.

## Conclusions

The diagnostic and therapeutic strategies for the management of cUGS are extremely various and usually require a multidisciplinary cooperation. Teams should include tropical diseases physicians, microbiologists, urologists, gynaecologists and radiologists. Currently available data force clinicians to adopt a case-by-case approach. However, we highlight some general points: (1) US is the first-line diagnostic for OU and *Schistosoma-*related bladder cancer; (2) PZQ can contribute to OU regression. Beneficial approaches in irreversible OU include a surgical approach using a Boari flap and a two ipsilateral double-J stenting after laser endoureterotomy. (3) No specific approaches for the *Schistosoma*-related bladder cancer were noted and as such it should be managed like bladder cancer due to other causes (4) Infertile patients with risk factors for *Schistosoma* infection should be screened by serology, and positive subjects should be treated. However, no data on the effect of PZQ on infertility were found. The conclusions are summarized in Table 5.

**Table 5:** Suggested diagnostic and management strategies of complicated urogenital schistosomiasis based on the literature search

Complication	Diagnostic strategy	Treatment strategy
Obstructive uropathy	Initial approach - Ultrasound Follow-on diagnostic test - CT scan /urography	Initial approach - Praziquantel In case of irreversible obstruction after praziquantel - Ureteral re-implantation with the Boari flap technique OR - Two ipsilateral double-J stent after laser endoureterotomy
*Schistosoma*-related bladder cancer	Initial approach - Ultrasound Follow-on diagnostic test - Cystoscopy with Trans Urethral Bladder Resection	Similar to non-*Schistosoma*-related bladder cancer (the patient should be also treated with praziquantel)
Infertility	Initial approach - *Schistosoma* serology	Initial approach - Praziquantel In case of irreversible infertility after praziquantel - Similar to non-*Schistosoma*-related infertility

### Supplementary Information

Below is the link to the electronic supplementary material.

Supplementary file1 (DOCX 169 KB)

## Data Availability

The datasets generated during the current study are available from the corresponding author on reasonable request.
