# Supplementary material for: Diagnosis and management of complicated urogenital schistosomiasis: a systematic review of the literature
Source: Infection. 2023 Jul 19;51(5):1185–221. doi: 10.1007/s15010-023-02060-5 (PMC10545601; doi:10.1007/s15010-023-02060-5)
Supplement: Supplementary file 1 — Supplementary file1 (DOCX 169 KB) [file 15010_2023_2060_MOESM1_ESM.docx]

**Search strategy**

This systematic review was reported in accordance with the indications of the Preferred Reporting Items for Systematic reviews and Meta-Analyses (PRISMA) Statement 2015 for the redaction of systematic reviews [1]. The MEDLINE (PubMed) search engine was used to conduct a search with the following query: (schistosomiasis[Title/Abstract] OR schistosoma[Title/Abstract] OR bilharziosis[Title/Abstract] OR bilharzia[Title/Abstract] OR bilharziasis[Title/Abstract] OR haematobium[Title/Abstract]) AND ((uropathy[Title/Abstract] OR hydronephrosis[Title/Abstract] OR hydroureteronephrosis[Title/Abstract] OR obstructive uropathy[Title/Abstract] OR renal impairment[Title/Abstract] OR renal failure[Title/Abstract] OR erectile dysfunction [Title/Abstract] OR kidney failure[Title/Abstract] OR urolithiasis[Title/Abstract] OR lithiasis[Title/Abstract] OR abortion[Title/Abstract] OR obsteric complications [Title/Abstract] OR ectopic pregnancy[Title/Abstract] OR infertility[Title/Abstract] OR sandy patches[Title/Abstract] OR cancer[Title/Abstract] OR tumor[Title/Abstract] OR neoplasm[Title/Abstract] OR ultrasonography[Title/Abstract] OR ultrasound[Title/Abstract] OR echography[Title/Abstract] OR ct scan[Title/Abstract] OR computed tomography[Title/Abstract] OR computer urography[Title/Abstract] OR magnetic resonance imaging[Title/Abstract] OR MRI[Title/Abstract] OR surgery[Title/Abstract] OR surgical[Title/Abstract] OR biopsy[Title/Abstract] OR resection[Title/Abstract] OR nephrectomy[Title/Abstract] OR stenting[Title/Abstract] OR stent[Title/Abstract] OR cystectomy [Title/Abstract] OR cystoscopy[Title/Abstract] OR cystourethroscopy[Title/Abstract] OR Colposcopy[Title/Abstract] OR colposcopic*[Title/Abstract] OR dialysis[Title/Abstract] OR chemotherapy[Title/Abstract])).

Articles were only included if published between January 1st 1990 and January 23rd 2021, which was the date of the last database search, based on the assumption that older therapies and diagnostic means would be still used in the last decades, if effective.

**Supplementary table 1** Studies regarding the diagnosis of UO in cUGS patients. Age refers to the whole cohort studied in the article (not just the complicated patients) when in italic.

| Ref. | Author | Year of publication | Publishing country | Country of infection | Study type | Patient type | Sex | Age | Diagnostic mean | Gold standard or comparator | n |
| --- | --- | --- | --- | --- | --- | --- | --- | --- | --- | --- | --- |
| [2] | Abdel-Wahab MF | 1992 | Egypt | Egypt | Transversal study | Endemic area residents | m/f | *range: [12-16]* | Egg count | Ultrasound | 422 |
| [3] | Abdel-Wahab MF | 1992 | Egypt | Egypt | Transversal study | Endemic area residents | m/f | mean [range]: 43.6 [15-62] (OU) | Ultrasound | Endovenous urography | 30 |
| [4] | Antwi S | 2014 | Ghana | Ghana | Case series | Endemic area residents | m/f | 7; 11; 12; 12; 12 | Ultrasound |  | 5 |
|  |  |  |  |  |  |  |  |  | Surgery |  | 2 |
|  |  |  |  |  |  |  |  |  | Anterograde urography |  | 1 |
|  |  |  |  |  |  |  |  |  | Endovenous urography |  | 1 |
| [5] | Badmos KB | 2009 | Nigeria | Nigeria | Case report | Endemic area residents | m | 25 | Ultrasound |  | 1 |
|  |  |  |  |  |  |  |  |  | Endovenous urography |  | 1 |
| [6] | Bakari AA | 2012 | Nigeria | Nigeria | Case report | Endemic area residents | m | 17 | Ultrasound |  | 1 |
|  |  |  |  |  |  |  |  |  | Endovenous urography |  | 1 |
| [7] | Bocanegra García C | 2018 | Angola | Angola | Cross-sectional study | Endemic area residents | m/f | *mean ± SD: 8.7 ± 3.2* | Ultrasound |  | 3 |
| [8] | Brouwer KC | 2003 | Zimbabwe | Zimbabwe | Transversal study | Endemic area residents | m/f | *range: [9-16]* | Parasite genetic profiling | Ultrasound | 25 |
| [9] | Brouwer KC | 2003 | Zimbabwe | Zimbabwe | Transversal study | Endemic area residents | m/f | *range: [9-16]* | Ultrasound |  | 67 |
| [10] | Dabo A | 1995 | Mali | Mali | Transversal study | Endemic area residents | m/f | *range: [6-15]* | Ultrasound |  | 307 |
| [11] | Fataar S | 1990 | Kuwait | Kuwait | Transversal study | Endemic area residents | m | mean [range]: 30 [21-46] | CT scan |  | 10 |
| [12] | Garba A | 1999 | Burkina Faso | Burkina Faso | Transversal study | Endemic area residents | m/f | *range: [7-15]* | Ultrasound |  | 390 |
| [13] | Ibrahim AI | 1991 | Saudi Arabia | Saudi Arabia | Transversal study | Endemic area residents | m/f | mean [range]: 34.6 [18-64] | Micturating cystourethrogram | Endovenous urography | 47 |
| [14] | Kazmi Z | 2020 | Pakistan | East Africa | Case report | Travelers | m | 24 | CT scan | Retrograde urography | 1 |
| [15] | Lorca J | 2019 | Spain | Myanmar | Case report | Travelers | f | 34 | Ultrasound | Biopsy | 1 |
|  |  |  |  |  |  |  |  |  | Uro-CT | Biopsy | 1 |
|  |  |  |  |  |  |  |  |  | Isotope renogram | Biopsy | 1 |
| [16] | Mandong BM | 2005 | Nigeria | Nigeria | Retrospective study | Endemic area residents | m/f | *[7-40]* | Surgery |  | 2 |
| [17] | Olajide AO | 2012 | Nigeria | Nigeria | Case report | Endemic area residents | m | 23 | Ultrasound | Surgery | 1 |
|  |  |  |  |  |  |  |  |  | Endovenous urography | Surgery | 1 |
| [18] | Oranusi CK | 2011 | Nigeria | Nigeria | Case report | Endemic area residents | m | 33 | Ultrasound | Surgery | 1 |
|  |  |  |  |  |  |  |  |  | Endovenous urography | Surgery | 1 |
| [19] | Pal PO | 2017 | UK | Zimbabwe | Case report | Migrants | m | 41 | CT scan | Ureteroscopy | 1 |
|  |  |  |  |  |  |  |  |  | Retrograde urography | Ureteroscopy | 1 |
|  |  |  |  |  |  |  |  |  | Isotope renogram | Surgery | 1 |
| [20] | Pallangyo P | 2020 | Tanzania | Tanzania | Case report | Endemic area residents | m | 38 | Clinical examination (palpable kidney) |  | 1 |
|  |  |  |  |  |  |  |  |  | Uro-CT |  | 1 |
|  |  |  |  |  |  |  |  |  | Urine filtration |  | 1 |
|  |  |  |  |  |  |  |  |  | Rectal biopsy |  | 1 |
| [21] | Pieras Ayala E | 2000 | Spain | North Africa | Case series | Migrants | m | 24 | Anterograde urography | Surgery | 1 |
| [22] | Pollock GR | 2020 | USA | Somalia | Case report | Migrants | m | 57 | CT scan |  | 1 |
| [23] | Rasendramino MH | 1998 | Madagascar | Madagascar | Transversal study | Endemic area residents | m/f | unspecified | Ultrasound |  | 436 |
| [24] | Remppis J | 2020 | Gabon | Gabon | Transversal study | Endemic area residents | m/f | unspecified | Ultrasound (clincian) | Ultrasound (radiologist) | 110 |
|  |  |  |  |  |  |  |  |  | Ultrasound (student) | Ultrasound (radiologist) | 104 |
| [25] | Richter J | 1996 | Ghana | Ghana | Transversal study | Endemic area residents | m/f | *median [range]: 14 [6-61]* | Ultrasound |  | 5 |
| [26] | Salah MA | 2000 | Yemen | Yemen | Transversal study | Endemic area residents | m/f | *mean [range]: 3.7 [0-14]* | Ultrasound |  | 158 |
| [27] | Salas-Coronas J | 2013 | Spain | Africa | Retrospective study | Migrants | m/f | *mean [range]: 26.7 [15-48]* | Ultrasound |  | 2 |
| [28] | Salas-Coronas J | 2020 | Spain | Africa | Retrospective study | Migrants | m | 23; 30; 31; 32; 33; 37; 46 | Ultrasound |  | 5 |
| [29] | Serieye J | 1996 | Madagascar | Madagascar | Transversal study | Endemic area residents | m/f | >5 | Ultrasound |  | 574 |
| [30] | Srougi V | 2017 | Brazil | Central Africa | Case report | Migrants | m | ~35 | CT scan |  | 1 |
| [31] | Vancauwenberghe T | 2013 | Belgium | Malawi | Case report | Travelers | f | 66 | CT scan | Surgery | 1 |
|  |  |  |  |  |  |  |  |  | Ureteroscopy | Surgery |  |
| [32] | Vester U | 1997 | Mali | Mali | Transversal study | Endemic area residents | m/f | >2 | Ultrasound |  | 532 |

**Supplementary table 2** Studies regarding the diagnosis of cUGS with bladder cancer. Age refers to the whole cohort studied in the article (not just the complicated patients) when in italic.

| Ref. | Author | Year of publication | Study country | Country of infection | Study type | Patient type | Sex | Age | Diagnostic mean | Gold standard or comparator | n |
| --- | --- | --- | --- | --- | --- | --- | --- | --- | --- | --- | --- |
| [33] | Abdel Mohsen MA | 1999 | Egypt | Egypt | Transversal study | Endemic area residents | m | mean ± SD: 54 ± 10 | Urinary nitrate | Biopsy | 61 |
|  |  |  |  |  |  |  |  |  | Urinary nitrite | Biopsy | 61 |
|  |  |  |  |  |  |  |  |  | Urinary apparent total N-nitroso compounds | Biopsy | 61 |
|  |  |  |  |  |  |  |  |  | Urinary N-nitrosodimethylamine | Biopsy | 61 |
|  |  |  |  |  |  |  |  |  | Urinary N-nitrosopiperidine | Biopsy | 61 |
|  |  |  |  |  |  |  |  |  | Urinary N-nitrosopyrrolidine | Biopsy | 61 |
|  |  |  |  |  |  |  |  |  | Urinary N-nitrosodibutylamine | Biopsy | 61 |
| [3] | Abdel-Wahab MF | 1992 | Egypt | Egypt | Transversal study | Endemic area residents | m/f | mean [range]: 60.4 [49-76] | Ultrasound | Endovenous urography | 30 |
| [34] | Ahmed NS | 2017 | Egypt | Egypt | Retrospective study | Endemic area residents | m/f | *range: [20-60]* | Biopsy |  | 11 |
| [35] | Ahmed SA | 1996 | Egypt | Egypt | Transversal study | Endemic area residents | unspecified | unspecified | Serum LDH | Biopsy | 63 |
|  |  |  |  |  |  |  |  |  | Serum aminoacidic pattern | Biopsy | 69 |
| [36] | Akinwale OP | 2008 | Nigeria | Nigeria | Transversal study | Endemic area residents | m/f | *mean [range]: 47.5 [40-70]* | Urine cytology |  | 32 |
| [37] | Al-Samawi AS | 2013 | Yemen | Yemen | Transversal study | Endemic area residents | m/f | *mean [range]: 57.6 [12-95]* | Biopsy |  | 31 |
| [38] | Alvarez Kindelan J | 1999 | Spain | Senegal | Case report | Migrants | m | 40 | Urine filtration | Biopsy | 1 |
|  |  |  |  |  |  |  |  |  | Urography | Biopsy | 1 |
|  |  |  |  |  |  |  |  |  | CT scan | Surgery | 1 |
| [39] | Amin HAA | 2019 | Egypt | Egypt | Transversal study | Endemic area residents | m/f | *mean [range]: 61.6 [20-78]* | Biopsy |  | 27 |
| [40] | Bedwani R | 1998 | Egypt | Egypt | Case-control study | Endemic area residents | m/f | *median [range]: 59 [21-74]* | Biopsy |  | 86 |
| [41] | Darré T | 2015 | Togo | Togo | Retrospective study | Endemic area residents | unspecified | unspecified | Biopsy or surgery |  | 54 |
| [42] | Eissa S | 2017 | Egypt | Egypt | Retrospective study | Endemic area residents | m/f | unspecified | Urinary ATG12 | Biopsy | 66 |
|  |  |  |  |  |  |  |  |  | Urinary FYCO1 | Biopsy | 66 |
|  |  |  |  |  |  |  |  |  | Urinary ULK1 | Biopsy | 66 |
|  |  |  |  |  |  |  |  |  | Urinary TECPR | Biopsy | 66 |
| [43] | Eissa S | 2015 | Egypt | Egypt | Retrospective study | Endemic area residents | unspecified | unspecified | Urinary miRNA-210 | Biopsy | 58 |
|  |  |  |  |  |  |  |  |  | Urinary miRNA-96 | Biopsy | 58 |
|  |  |  |  |  |  |  |  |  | Urinary lncRNA-UCA1 | Biopsy | 58 |
|  |  |  |  |  |  |  |  |  | Hyaluronidase mRNA in urine | Biopsy | 58 |
| [44] | Eissa S | 2007 | Egypt | Egypt | Retrospective study | Endemic area residents | m/f | *mean ± SD [range]: 57 ± 12 [25-82]* | Urinary MMP-2 | Biopsy | 224 |
|  |  |  |  |  |  |  |  |  | Urinary MMP-9 | Biopsy | 224 |
|  |  |  |  |  |  |  |  |  | Urinary TIMP-2 | Biopsy | 224 |
|  |  |  |  |  |  |  |  |  | Urinary MMP-2/TIMP-2 | Biopsy | 224 |
|  |  |  |  |  |  |  |  |  | Urinary MMP-9/TIMP-2 | Biopsy | 224 |
| [45] | Eissa S | 2013 | Egypt | Egypt | Retrospective study | Endemic area residents | m/f | *mean ± SD [range]: 60.1 ± 11.8 [26-83]* | Urinary survivin | Biopsy | 100 |
|  |  |  |  |  |  |  |  |  | Hyaluronidase mRNA in urine | Biopsy | 100 |
| [46] | Eissa S | 2015 | Egypt | Egypt | Retrospective study | Endemic area residents | m/f | *mean ± SD [range]: 58.8 ± 11.6 [28-85]* | Urinary miRNA-96 | Biopsy | 47 |
| [47] | Eissa S | 2005 | Egypt | Egypt | Retrospective study | Endemic area residents | m/f | *mean ± SD [range]: 58.0 ± 10.2 [35-82]* | Hyaluronidase mRNA in urine | Biopsy | 274 |
|  |  |  |  |  |  |  |  |  | Urinary CK-20 mRNA | Biopsy | 274 |
| [48] | Eissa S | 2004 | Egypt | Egypt | Retrospective study | Endemic area residents | m/f | *mean ± SD [range]: 55.9 ± 8.8 [40-78]* | Urinary CK-20 mRNA | Biopsy | 131 |
|  |  |  |  |  |  |  |  |  | Urinary angiogenin | Biopsy | 131 |
| [49] | Eissa S | 2003 | Egypt | Egypt | Retrospective study | Endemic area residents | unspecified | *mean [range]: 58 [30-48]* | Telomerase activity in urine | Biopsy | 100 |
|  |  |  |  |  |  |  |  |  | Urinary MMP-9 | Biopsy | 95 |
| [50] | Eissa S | 2015 | Egypt | Egypt | Retrospective study | Endemic area residents | m/f | *mean ± SD [range]: 61.8 ± 8.2 [40–80]* | Urinary lncRNA-UCA1 | Biopsy | 56 |
| [51] | Eissa S | 2015 | Egypt | Egypt | Retrospective study | Endemic area residents | unspecified | unspecified | Urinary miRNA-210 | Biopsy | 96 |
|  |  |  |  |  |  |  |  |  | Urinary miRNA-10b | Biopsy | 96 |
|  |  |  |  |  |  |  |  |  | Urinary miRNA-29c | Biopsy | 96 |
| [52] | Eissa S | 2014 | Egypt | Egypt | Retrospective study | Endemic area residents | m/f | *mean ± SD [range]: 52 ± 10 [25-79]* | Urinary HURP mRNA | Biopsy | 151 |
| [53] | Eissa S | 2013 | Egypt | Egypt | Retrospective study | Endemic area residents | m/f | *mean ± SD [range]: 58.1 ± 10.2 [42-78]* | Urinary hTERT mRNA | Biopsy | 105 |
|  |  |  |  |  |  |  |  |  | Urinary scatter factor | Biopsy | 105 |
| [54] | Eissa S | 2007 | Egypt | Egypt | Retrospective study | Endemic area residents | m/f | *mean ± SD [range]: 57.8 ± 10 [36-78]* | Telomerase activity in urine | Biopsy | 243 |
|  |  |  |  |  |  |  |  |  | Urinary telomerase mRNA | Biopsy | 243 |
|  |  |  |  |  |  |  |  |  | Urinary hTERT mRNA | Biopsy | 243 |
| [55] | Eissa S | 2002 | Egypt | Egypt | Retrospective study | Endemic area residents | unspecified | *mean [range]: 56.5 [30-76]* | Urinary NMP22 | Biopsy | 215 |
|  |  |  |  |  |  |  |  |  | Urinary fibronectin | Biopsy | 215 |
|  |  |  |  |  |  |  |  |  | Urinary UBC | Biopsy | 215 |
| [56] | Eissa S | 2008 | Egypt | Egypt | Retrospective study | Endemic area residents | unspecified | *mean ± SD [range]: 56.6 ± 9.6 [36-80]* | Urinary CD44 | Biopsy | 156 |
|  |  |  |  |  |  |  |  |  | Urinary CK-20 mRNA | Biopsy | 156 |
| [57] | el-Ahmady O | 1999 | Egypt | Egypt | Transversal study | Endemic area residents | m/f | *range: [35-78]* | Urinary CYFRA21-1 | Biopsy | 270 |
| [58] | El-Sharkawi F | 2014 | Egypt | Egypt | Retrospective study | Endemic area residents | m/f | mean [range]: 62.8 [38-84] | Urinary MMP-9 | Biopsy | 82 |
|  |  |  |  |  |  |  |  |  | Urinary MMP-3 | Biopsy | 82 |
| [11] | Fataar S | 1990 | Kuwait | Kuwait | Transversal study | Endemic area residents | m | mean [range]: 30 [21-46] | CT scan |  | 10 |
| [59] | Gaber DA | 2020 | Egypt | Egypt | Transversal study | Endemic area residents | m/f | mean ± SD [range]: 61 ± 10 [44-75] | Sha-miR-71a gene in urine | IHA | 50 |
|  |  |  |  |  |  |  |  |  | Urinary MAPK3 | IHA | 85 |
|  |  |  |  |  |  |  |  |  | Urinary MAPK3 mRNA | IHA | 85 |
| [60] | Gaye AM | 2016 | Senegal | Senegal | Retrospective study | Endemic area residents | unspecified | unspecified | Biopsy or surgery |  | 31 |
| [61] | Groeneveld AE | 1996 | Burkina Faso | Burkina Faso | Retrospective study | Endemic area residents | m/f | *mean: 62.1* | Biopsy |  | 197 |
| [62] | Ketabchi A | 2012 | Iran | Iran | Case report | Travelers | m | 60 | Ultrasound | Biopsy | 1 |
| [63] | Khaled HM | 2001 | Egypt | Egypt | Transversal study | Endemic area residents | unspecified | *mean [range]: 57 [26-85]* | BTA stat test | Biopsy | 85 |
|  |  |  |  |  |  |  |  |  | BTA TRAK assay | Biopsy | 85 |
| [64] | Lodhia J | 2020 | Tanzania | Tanzania | Case series | Endemic area residents | f | 53 | Ultrasound | Biopsy | 1 |
| [65] | Marbjerg LH | 2015 | Denmark | Mozambique | Case report | Migrants | f | 40 | CT scan | Biopsy | 1 |
|  |  |  |  |  |  |  |  |  | CT scan-PET | Biopsy | 1 |
| [66] | Martin JW | 2018 | Egypt | Egypt | Retrospective study | Endemic area residents | m/f | *mean [range]: 54 [20-87]* | Surgery |  | 802 |
| [67] | Mina SN | 2015 | Egypt | Egypt | Case report | Endemic area residents | m | 80 | Cystoscopy | TURB | 1 |
|  |  |  |  |  |  |  |  |  | CT scan | TURB | 1 |
| [68] | Mohammed MA | 2013 | Egypt | Egypt | Retrospective study | Endemic area residents | m/f | *mean ± SD: 54 ± 8* | Urinary MMP-2 | Biopsy | 166 |
|  |  |  |  |  |  |  |  |  | Urinary MMP-9 | Biopsy | 166 |
|  |  |  |  |  |  |  |  |  | Urinary MMP-9/NGAL | Biopsy | 166 |
|  |  |  |  |  |  |  |  |  | MMP-9 dimers in urine | Biopsy | 166 |
|  |  |  |  |  |  |  |  |  | Urinary MMP-9/TIMP | Biopsy | 166 |
|  |  |  |  |  |  |  |  |  | Urinary ADAMTS-7 | Biopsy | 166 |
| [69] | Mourad WA | 1998 | Saudi Arabia | Saudi Arabia | Case report | Endemic area residents | m | 52 | Cystoscopy | Biopsy | 1 |
|  |  |  |  |  |  |  |  |  | CT scan | Biopsy | 1 |
| [70] | Mungadi IA | 2007 | Nigeria | Nigeria | Retrospective study | Endemic area residents | m/f | *mean [range]: 46.0 [20-82]* | Biopsy |  | 16 |
| [71] | Saied GM | 2007 | Egypt | Egypt | Retrospective study | Endemic area residents | unspecified | unspecified | Urinary CEA | Surgery | 32 |
|  |  |  |  |  |  |  |  |  | Serum CEA | Surgery | 32 |
| [72] | Santos J | 2015 | Angola | Angola | Transversal study | Endemic area residents | m/f | median [range]: 41 [3-75] | Ultrasound | Cystoscopy | 80 |
| [30] | Srougi V | 2017 | Brazil | Central Africa | Case report | Migrants | m | ~35 | CT scan | Biopsy | 1 |
| [73] | Vieira P | 2007 | Portugal | Mozambique | Case series | Expatriates | m | 60 | Biopsy |  | 1 |
|  |  |  |  |  |  |  |  |  | Urine filtration |  | 1 |
| [74] | Yang H | 2005 | Egypt | Egypt | Retrospective study | Endemic area residents | unspecified | unspecified | Serum HPV-16 DNA | Biopsy | 27 |
|  |  |  |  |  |  |  |  |  | Urinary HPV-16 DNA | Biopsy | 24 |

**Supplementary Table 3** Studies regarding the diagnosis of cUGS with infertility and ectopic pregnancy. ^a^ haematuria and/or ultrasound and/or urine filtration. Age refers to the whole cohort studied in the article (not just the complicated patients) when in italic.

| Ectopic pregnancy | | | | | | | | | | |
| --- | --- | --- | --- | --- | --- | --- | --- | --- | --- | --- |
| Ref. | Author | Year of publication | Publishing country | Country of infection | Study type | Patient type | Age | Diagnostic mean | Gold standard or comparator | n |
| [75] | Bahrami S | 2006 | USA | East Africa | Case report | Migrants | 20 | β-hCG | Surgery | 1 |
|  |  |  |  |  |  |  |  | Ultrasound | Surgery | 1 |
|  |  |  |  |  |  |  |  | Urine filtration | Surgery | 1 |
| [76] | Bugalho A | 1991 | Mozambique | Mozambique | Case series | Endemic area residents | 21; 22; 28; 33 | Surgery |  | 4 |
| [77] | Ekoukou D | 1995 | France | Mali | Case series | Migrants | 29 | *β*-hCG | Surgery | 1 |
|  |  |  |  |  |  |  |  | Ultrasound | Surgery | 1 |
| [78] | Eogan M | 2002 | Ireland | Nigeria | Case report | Migrants | 31 | Ultrasound | Surgery | 1 |
| [79] | Garba M | 2004 | Niger | Niger | Case report | Endemic area residents | 21 | Ultrasound | Surgery | 1 |
| [80] | Laroche J | 2016 | France | Mali | Case report | Travelers | 26 | Ultrasound |  | 1 |
|  |  |  |  |  |  |  |  | *β*-hCG |  | 1 |
|  |  |  |  |  |  |  |  | Urine filtration | Surgery | 1 |
| [81] | Laxman VV | 2008 | UK | Zambia | Case report | Migrants | 37 | Ultrasound |  | 1 |
|  |  |  |  |  |  |  |  | *β*-hCG |  | 1 |
| [82] | Nouhou H | 1998 | Niger | Niger | Retrospective study | Endemic area residents | mean [range]: 30 [17-70] | Surgery |  | 1 |
| [83] | Odubamowo KH | 2014 | Nigeria | Nigeria | Case report | Endemic area residents | 32 | Ultrasound | Surgery | 1 |
| [84] | Okonofua FE | 1990 | Nigeria | Nigeria | Case report | Endemic area residents | 34 | Surgery |  | 1 |
| [85] | Sahu L | 2013 | India | unknown | Case report | Travelers | 25 | *β*-hCG |  | 1 |
|  |  |  |  |  |  |  |  | Ultrasound |  | 1 |
| [86] | Schneider D | 2000 | South Africa | South Africa | Case report | Endemic area residents | 35 | *β*-hCG |  | 1 |
|  |  |  |  |  |  |  |  | Ultrasound |  | 1 |
| [87] | Ville Y | 1991 | Gabon | Gabon | Case series | Endemic area residents | 26 | *β*-hCG | Surgery | 1 |
|  |  |  |  |  |  |  |  | Ultrasound | Surgery | 1 |
| Female infertility | | | | | | | | | | |
| Ref. | Author | Year of publication | Publishing country | Country of infection | Study type | Patient type | Age | Diagnostic mean | Gold standard or comparator | n |
| [88] | Bailey SL | 2011 | UK | Malawi | Case series | Travelers | 43 | Surgery |  | 1 |
| [89] | Balasch J | 1995 | Spain | Nigeria | Case report | Migrants | 26 | Hysterosalpingography | Surgery | 1 |
|  |  |  |  |  |  |  |  | Ultrasound | Surgery | 1 |
| [90] | Darwish AM | 1999 | Egypt | Egypt | Case series | Endemic area residents | 26; 30; 30 | Serology | Biopsy | 3 |
|  |  |  |  |  |  |  |  | Urine filtration | Biopsy | 3 |
| [77] | Ekoukou D | 1995 | France | Mali | Case series | Migrants | 29 | Hysteroscopy | Surgery | 1 |
|  |  |  |  |  |  |  |  | Urine filtration | Biopsy | 1 |
| [91] | Jones KD | 2003 | UK | South Africa | Case report | Travelers | 33 | Urine filtration | Biopsy | 1 |
|  |  |  |  |  |  |  |  | ELISA | Biopsy | 1 |
| [92] | Kjetland EF | 1996 | Malawi | Malawi | Transversal study | Endemic area residents | *median [range]: 22 [15-47]* | Multiple biopsies |  | 31 |
| [93] | Kjetland EF | 2010 | Zimbabwe | Zimbabwe | Transversal study | Endemic area residents | *range: [20-49]* | Cervical smear |  | 23 |
| [94] | Krolikowski A | 1995 | South Africa | South Africa | Case report | Endemic area residents | 27 | Hysterosalpingography | Surgery | 1 |
| [95] | Morice P | 1993 | France | unknown | Case report | Migrants | 33 | Hysterosalpingography | Surgery | 1 |
| [96] | Nayama M | 2007 | Niger | Niger | Transversal study | Endemic area residents | *mean [range]: 26 [15-40]* | Cervical smear |  | 42 |
|  |  |  |  |  |  |  |  | Biopsy |  | 26 |
|  |  |  |  |  |  |  |  | Pelvic pain | Variousa | 42 |
|  |  |  |  |  |  |  |  | Leucorrhea | Variousa | 42 |
|  |  |  |  |  |  |  |  | Genital itch | Variousa | 42 |
|  |  |  |  |  |  |  |  | Dyspareunia | Variousa | 42 |
|  |  |  |  |  |  |  |  | Hysterosalpingography |  | 11 |
| [82] | Nouhou H | 1998 | Niger | Niger | Retrospective study | Endemic area residents | mean [range]: 30 [17-70] | Biopsy |  | 6 |
| [97] | Owusu-Bempah A | 2013 | Ghana | Ghana | Case report | Endemic area residents | 34 | Ultrasound | Surgery | 1 |
|  |  |  |  |  |  |  |  | Hysterosalpingography | Surgery | 1 |
| [98] | Richter J | 1995 | Malawi | Malawi | Transversal study | Endemic area residents | *median [range]: 27 [17-36]* | Ultrasound |  | 2 |
| [99] | Santos J | 2014 | Angola | Angola | Transversal study | Endemic area residents | mean [range]: 25.6 [18–41] | Catechol-estrogens/ DNA adducts in urine | Urine filtration | 29 |
| [100] | Schanz A | 2010 | Germany | Nigeria | Case report | Migrants | 30 | Ultrasound | Surgery | 1 |
|  |  |  |  |  |  |  |  | CT scan | Surgery | 1 |
| [101] | Swai B | 2006 | Mali | Tanzania | Retrospective study | Endemic area residents | *median [range]: 34 [5-61]* | Biopsy |  | 7 |
| [102] | Van Den Broucke S | 2020 | Belgium | Mali | Case report | Travelers | 28 | Biopsy |  | 1 |
|  |  |  |  |  |  |  |  | ELISA |  | 1 |
|  |  |  |  |  |  |  |  | IHA |  | 1 |
|  |  |  |  |  |  |  |  | PCR |  | 1 |
| Male infertility | | | | | | | | | | |
| Ref. | Author | Year of publication | Publishing country | Country of infection | Study type | Patient type | Age | Diagnostic mean | Gold standard or comparator | n |
| [103] | Al-Qahtani SM | 2010 | France | Egypt | Case report | Migrants | 31 | Ultrasound | Surgery | 1 |
| [104] | Kini S | 2009 | UK | Uganda | Case report | Travelers | unspecified | Ultrasound |  | 1 |
|  |  |  |  |  |  |  |  | Gonadotropins |  | 1 |
|  |  |  |  |  |  |  |  | Testosterone |  | 1 |

**Supplementary Table 4** Case reports regarding cUGS that required a biopsy or an invasive procedure but did not manifest any other complications and responded well to treatment with PZQ.

| Ref. | Author | Year of publication | Study country | Country of infection | Patient type | Sex | Age | Lesion | n | Invasive procedure | Diagnostic mean | Positive result? |
| --- | --- | --- | --- | --- | --- | --- | --- | --- | --- | --- | --- | --- |
| [105] | Alvarez Maestro M | 2010 | Spain | Gambia | Migrants | m | 21 | Bladder mass | 1 | Biopsy | Ultrasound | Yes |
|  |  |  |  |  |  |  |  |  |  |  | Endovenous urography | No |
| [106] | Al-Saeed O | 2003 | Kuwait | Kuwait | Endemic area residents | m | 33; 41; 41; 45 | Seminal vesicle mass | 4 | FNA or biopsy | Ultrasound | Yes |
| [107] | Aytaç B | 2012 | Turkey | Central Africa | Travelers | m | 37 | Bladder mass | 1 | Biopsy | Ultrasound | Yes |
|  |  |  |  |  |  |  |  |  |  |  | Urine filtration | Yes |
| [108] | Azami MA | 2018 | Morocco | Morocco | Endemic area residents | f | 28 | Infected ovarian cyst | 1 | Laparotomy | Ultrasound | Yes |
|  |  |  |  |  |  |  |  |  |  |  | CT scan | Yes |
|  |  |  |  |  |  |  |  |  |  |  | Urine filtration | No |
| [109] | Badmus TA | 2012 | Nigeria | Nigeria | Endemic area residents | m | 16 | Testicular mass | 1 | Orchiectomy | Ultrasound | Yes |
| [88] | Bailey SL | 2011 | UK | Malawi | Travelers | f | 34 | Infected ovarian cyst | 1 | Surgery | Ultrasound | Yes |
| [110] | Ballesta Martínez B | 2019 | Spain | Various African countries | Migrants | m | 29 | Bladder mass | 1 | Biopsy | Ultrasound | Yes |
|  |  |  |  |  |  |  |  |  |  |  | Urine filtration | Yes |
| [111] | Carrión López P | 2010 | Spain | Mali | Migrants | m | 19 | Pseudopolyp | 1 | Biopsy | Ultrasound | Yes |
|  |  |  |  |  |  |  |  |  |  |  | Urine filtration | Yes |
| [112] | Chahdi H | 2018 | Morocco | Mauritania | Travelers | m | 25 | Haematuria | 1 | Biopsy | Urine filtration | Yes |
| [90] | Darwish AM | 1999 | Egypt | Egypt | Endemic area residents | f | 34 | Tubal mass | 1 | Minilaparotomy | Urine filtration | Yes |
|  |  |  |  |  |  |  |  |  |  |  | Serology | Yes |
| [113] | De NV | 2019 | Vietnam | Angola | Expatriates | m | 43 | Bladder mass | 1 | Laparoscopy | Urine filtration | Yes |
|  |  |  |  |  |  |  |  |  |  |  | PCR of urine | Yes |
| [114] | Dessyn JF | 2016 | France | Madagascar | Travelers | f | 25 | Pseudopolyp | 1 | Biopsy | Ultrasound | Yes |
|  |  |  |  |  |  |  |  |  |  |  | Serology | Yes |
| [115] | Dzeing-Ella A | 2009 | France | Mali | Travelers | f | 34 | Cervical dysplasia | 1 | Biopsy | Urine filtration | Yes |
| [116] | Efared B | 2018 | Morocco | Morocco | Endemic area residents | f | 51 | Ovarian mass | 1 | Surgery | Surgery | Yes |
| [117] | Fabiano M | 2020 | Italy | Africa | Migrants | m | 11 | Bladder mass | 1 | Biopsy | Ultrasound | Yes |
|  |  |  |  |  |  |  |  |  |  |  | CT scan | Yes |
|  |  |  |  |  |  |  |  |  |  |  | Urine filtration | No |
| [118] | Fall I | 1992 | Senegal | Senegal | Endemic area residents | m | 9 | Testicular mass | 1 | Orchiectomy | Ultrasound | Yes |
| [119] | Haghighi L | 2020 | Iran | Iran | Travelers | m | 25 | Bladder granulomas | 1 | Biopsy | Ultrasound | Yes |
| [120] | Hosny K | 2018 | UK | Malawi | Travelers | m | 65 | Bladder granulomas | 1 | Biopsy | Ultrasound | Yes |
|  |  |  |  |  |  |  |  |  |  |  | CT scan | Yes |
|  |  |  |  |  |  |  |  |  |  |  | Urine filtration | Yes |
| [121] | Kato-Hayashi N | 2013 | Japan | Mali | Travelers | m | 21 | Infection not responsive to PZQ | 1 | Biopsy | Urine filtration | Yes |
|  |  |  |  |  |  |  |  |  |  |  | Cell-free schistosome DNA | Yes |
| [122] | Kameh D | 2004 | USA | South Africa | Migrants | f | 37 | Female genital schistosomiasis | 1 | Biopsy | Cervical smear | Yes |
|  |  |  |  |  |  |  |  |  |  |  | Urine filtration | No |
|  |  |  |  |  |  |  |  |  |  |  | Stool microscopy | No |
| [123] | Kohno M | 2008 | Japan | Africa | Travelers | m | 31 | Bladder granulomas | 1 | Biopsy | CT scan | Yes |
|  |  |  |  |  |  |  |  |  |  |  | MRI | Yes |
| [94] | Krolikowski A | 1995 | South Africa | South Africa | Endemic area residents | f | 27 | Asherman syndrome | 1 | Laparoscopy | Hysterosalpingography | Yes |
| [124] | Labairu Huerta L | 2007 | Spain | Central Africa | Migrants | m | 26 | Bladder mass | 1 | Biopsy | Biopsy only |  |
| [125] | Lee Y | 2020 | South Korea | Kenya or Malawi | Expatriates | f | 23 | Bladder granulomas | 1 | Biopsy | CT scan | Yes |
|  |  |  |  |  |  |  |  |  |  |  | PCR of urine | Yes |
|  |  |  |  |  |  |  |  |  |  |  | Serology | Yes |
| [126] | López López AI | 2007 | Spain | Various African countries | Migrants | m | 28 | Ureteral stenosis | 1 | Biopsy | Urine filtration | No |
|  |  |  |  |  |  |  |  |  |  |  | Serology | Yes |
|  |  |  |  |  |  |  |  |  |  |  | X-rays | Yes |
|  |  |  |  |  |  |  |  |  |  |  | Ultrasound | No |
|  |  |  |  |  |  |  |  |  |  |  | Endovenous urography | Yes |
| [127] | Mascarenhas A | 2011 | Portugal | Guinea-Bissau | Migrants | m | 10 | Bladder granulomas | 1 | Biopsy | Ultrasound | Yes |
|  |  |  |  |  |  |  |  |  |  |  | Urine filtration | Yes |
| [128] | Neal PM | 2004 | USA | Somalia | Migrants | m | 32 | Hydroureter | 1 | Biopsy | Endovenous urography | Yes |
|  |  |  |  |  |  |  |  |  |  |  | Retrograde urography | Yes |
|  |  |  |  |  |  |  |  |  |  |  | Urine filtration | No |
| [129] | Oguntunde OA | 2020 | Nigeria | Nigeria | Endemic area residents | m | 9 | Testicular mass | 1 | Surgical exploration | Cystoscopy | Yes |
| [130] | Pedalino M | 2010 | Italy | Africa | Travelers | m | 34 | Bladder granulomas | 1 | Biopsy | Ultrasound | No |
| [131] | Pinto SZ | 2019 | South Africa | South Africa | Endemic area residents | f | 10 | Pseudopolyp | 1 | Biopsy | Urine filtration | No |
|  |  |  |  |  |  |  |  |  |  |  | Ultrasound | Yes |
| [132] | Rambau PF | 2011 | Tanzania | Tanzania | Endemic area residents | m | 9 | Testicular swelling and atrophy | 1 | Orchiectomy | Urine filtration | No |
| [133] | Samuel MI | 2015 | UK | Malawi | Travelers | f | 29 | Lesion of labia minora | 1 | Biopsy | Urine filtration | Yes |
| [134] | Scarlata F | 2005 | Italy | Ghana | Migrants | m | 26 | Bladder granulomas | 1 | Biopsy | Urine filtration | Yes |
|  |  |  |  |  |  |  |  |  |  |  | Stool microscopy | No |
|  |  |  |  |  |  |  |  |  |  |  | Serology | Yes |
| [135] | Silva IM | 2006 | Brazil | Mozambique | Travelers | m | median [range]: 29 [26-36] | Infection not responsive to PZQ | 25 | Biopsy | Cystoscopy | Yes (22/25) |
| [136] | Silva IM | 2008 | Brazil | Mozambique | Travelers | m | unspecified | Infection not responsive to PZQ | 1 | Biopsy | Urine filtration | Yes |
|  |  |  |  |  |  |  |  |  |  |  | Cystoscopy | Yes |
| [137] | Soans B | 1999 | Australia | Malawi | Travelers | m | 33 | Testicular mass | 1 | Orchiectomy | Ultrasound | Yes |
|  |  |  |  |  |  |  |  |  |  |  | Testis tumor markers | No |
|  |  |  |  |  |  |  |  |  |  |  | Urine filtration | No |
|  |  |  |  |  |  |  |  |  |  |  | Serology | Yes |
| [138] | Sultana SR | 1995 | UK | Malawi | Travelers | f | 22 | Bladder granulomas | 1 | Biopsy | Ultrasound | No |
|  |  |  |  |  |  |  |  |  |  |  | X-rays | No |
| [139] | Tan WP | 2017 | USA | Mozambique | Migrants | m | 22 | Bladder granulomas | 1 | Biopsy | Biopsy only |  |
| [140] | Tilli M | 2019 | Italy | Subsaharian Africa | Migrants | unspecified | unspecified | Infection not responsive to PZQ | 7 | Biopsy | Cystoscopy | Yes (all 7) |
| [141] | Torricelli M | 1998 | Italy | Morocco | Migrants | m | 14 | Pseudopolyp | 1 | Cystotomy | Ultrasound | Yes |
|  |  |  |  |  |  |  |  |  |  |  | Urine filtration | Yes |
| [142] | Turkistani I | 2002 | Saudi Arabia | Saudi Arabia | Endemic area residents | f | 37 | Infestation of a paravaginal tumor | 1 | Laparotomy | RMN | Yes |
|  |  |  |  |  |  |  |  |  |  |  | Urine filtration | No |
|  |  |  |  |  |  |  |  |  |  |  | Stool microscopy | No |
|  |  |  |  |  |  |  |  |  |  |  | Serology | Yes |
| [143] | Ze Ondo C | 2013 | Senegal | Senegal | Endemic area residents | m | 6; 38 | Testicular mass | 2 | Orchiectomy | Urine filtration | No (both) |
|  |  |  |  |  |  |  |  |  |  |  | Ultrasound | Yes |
|  |  |  |  |  |  |  |  |  |  |  | Testis tumor markers | No |
| [144] | Zepeda CM | 2015 | USA | Egypt | Migrants | m | 65 | Bladder granulomas | 1 | Biopsy | Urine filtration | No |

**Supplementary Table 5** Studies regarding the treatment of cUGS with hydronephrosis. Age refers to the whole cohort studied in the article (not just the complicated patients) when in italic.

| Ref. | Author | Year of publication | Study country | Country of infection | Study type | Patient type | Sex | Age | n | Treatment type | Follow-up length |
| --- | --- | --- | --- | --- | --- | --- | --- | --- | --- | --- | --- |
| [4] | Antwi S | 2014 | Ghana | Ghana | Case series | Endemic area residents | m/f | 7; 12; 12 | 3 | Surgery | ongoing |
|  |  |  |  |  |  |  | m | 12 | 1 | PZQ only | 1 year |
| [5] | Badmos KB | 2009 | Nigeria | Nigeria | Case report | Endemic area residents | m | 25 | 1 | Surgery | 6 months |
| [6] | Bakari AA | 2012 | Nigeria | Nigeria | Case report | Endemic area residents | m | 17 | 1 | Surgery | 1 year |
| [145] | Garba A | 2004 | Niger | Niger | Prospective cohort | Endemic area residents | m/f | unspecified | 1905 | MDA (PZQ) | 3 years |
| [146] | Hatz C | 1990 | Tanzania | Tanzania | Prospective cohort | Endemic area residents | unspecified | *mean [range]: 12.5 [7-20]* | 2 | PZQ only | 6 months |
| [147] | Kardorff R | 1994 | Mali | Mali | Prospective cohort | Endemic area residents | m/f | unspecified | 9 | PZQ only | 1 year |
| [14] | Kazmi Z | 2020 | Pakistan | East Africa | Case report | Travelers | m | 24 | 1 | Combined | 3 months |
| [148] | King CH | 1990 | Kenya | Kenya | Prospective cohort | Endemic area residents | m/f | *range: [4-21]* | 8 | Metrifonate | 1 year |
|  |  |  |  |  |  |  |  |  | 5 | PZQ only | 1 year |
| [149] | King CH | 2002 | Kenya | Kenya | RCT | Endemic area residents | m/f | *range: [4-23]* | 9 | 20 mg/kg PZQ | 9 months |
|  |  |  |  |  |  |  |  |  | 14 | 40 mg/kg PZQ | 9 months |
| [15] | Lorca J | 2019 | Spain | Myanmar | Case report | Travelers | f | 34 | 1 | Surgery | 1 year |
| [150] | Mohyelden K | 2020 | Egypt | Egypt | RCT | Endemic area residents | m/f | mean ± SD: 43.6 ± 13.0 | 35 | Combined | at least 18 months |
|  |  |  |  |  |  |  |  |  | 35 | Combined | at least 18 months |
| [17] | Olajide AO | 2012 | Nigeria | Nigeria | Case report | Endemic area residents | m | 23 | 1 | Surgery | 3 years |
| [18] | Oranusi CK | 2011 | Nigeria | Nigeria | Case report | Endemic area residents | m | 33 | 1 | Surgery | 6 months |
| [151] | Ouma JH | 2005 | Kenya | Kenya | Case-control study | Endemic area residents | m/f | *mean ± SD: 29.1 ± 7.5 (age at follow-up)* | 132 | MDA (PZQ,  metrifonate) | 10-18 years |
| [19] | Pal PO | 2017 | UK | Zimbabwe | Case report | Migrants | m | 41 | 1 | Combined | 15 weeks |
| [21] | Pieras Ayala E | 2000 | Spain | North Africa | Case series | Migrants | m | 24 | 1 | Combined |  |
| [22] | Pollock GR | 2020 | USA | Somalia | Case report | Migrants | m | 57 | 1 | Robotic |  |
| [152] | Rasendramino MH | 1998 | Madagascar | Madagascar | Prospective cohort | Endemic area residents | m/f | >5 | 574 | MDA (PZQ) | 1 year |
| [153] | Ravi G | 1993 | Saudi Arabia | Saudi Arabia | Prospective cohort | Endemic area residents | unspecified | mean [range]: 35 [4-80] | 150 | Surgery | 6-24 months |
| [28] | Salas-Coronas J | 2020 | Spain | Africa | Retrospective study | Migrants | m | 23; 31 | 2 | Surgery |  |
|  |  |  |  |  |  |  |  | 30 | 1 | PZQ only |  |
| [154] | Subramanian AK | 1999 | Kenya | Kenya | Prospective cohort | Endemic area residents | m/f | *median [range]: 24 [16-35] (age at follow-up)* | 517 | MDA (PZQ,  metrifonate) | 13 years |
| [140] | Tilli M | 2019 | Italy | Subsaharian Africa | Retrospective study | Migrants | unspecified | unspecified | 1 | Surgery |  |
| [31] | Vancauwenberghe T | 2013 | Belgium | Malawi | Case report | Travelers | f | 66 | 1 | Surgery |  |

**Supplementary Table 6** Studies regarding the treatment of cUGS with bladder cancer.

| Ref. | Author | Year of publication | Study country | Country of infection | Study type | Patient type | Sex | Age | n | Treatment type | Follow-up length |
| --- | --- | --- | --- | --- | --- | --- | --- | --- | --- | --- | --- |
| [155] | Abdou A | 2012 | France | Africa | Retrospective study | Travelers and migrants | m/f | mean [range]: 50 [37-65] | 15 | Surgery | 2-10 years |
| [156] | Almeida M | 2014 | Portugal | Africa | Case report | Migrants | m | 62 | 1 | Combined | 19 months |
| [38] | Alvarez Kindelan J | 1999 | Spain | Senegal | Case report | Migrants | m | 40 | 1 | Surgery |  |
| [157] | Aly MS | 2012 | Egypt | Egypt | Prospective cohort | Endemic area residents | m/f | mean ± SD; median [range]: 53.9 ± 9.5; 54.5 [39-77] | 41 | Chemotherapy |  |
| [62] | Ketabchi A | 2012 | Iran | Iran | Case report | Travelers | m | 60 | 1 | Combined | 7 months |
| [64] | Lodhia J | 2020 | Tanzania | Tanzania | Case series | Endemic area residents | f | 53 | 1 | Radiotherapy |  |
| [65] | Marbjerg LH | 2015 | Denmark | Mozambique | Case report | Migrants | f | 40 | 1 | Surgery |  |
| [67] | Mina SN | 2015 | Egypt | Egypt | Case report | Endemic area residents | m | 80 | 1 | Endoscopy |  |
| [69] | Mourad WA | 1998 | Saudi Arabia | Saudi Arabia | Case report | Endemic area residents | m | 52 | 1 | Chemotherapy | ongoing |
| [28] | Salas-Coronas J | 2020 | Spain | Africa | Retrospective study | Migrants | m | 37; 46 | 2 | Surgery |  |
| [30] | Srougi V | 2017 | Brazil | Central Africa | Case report | Migrants | m | ~35 | 1 | Surgery | 49 days |
| [73] | Vieira P | 2007 | Portugal | Mozambique | Case series | Expatriates | m | 60 | 1 | Combined |  |
| [158, 159] | Wishahi MM | 1994 | Egypt | Egypt | Prospective cohort | Endemic area residents | m | mean ± SD; median [range]: 42.1 ± 10.0; 43 [27-59] | 13 | Immunotherapy | 6-20 months |
| [159] | Wishahi MM | 1995 | Egypt | Egypt | Prospective cohort | Endemic area residents | m | mean ± SD; median [range]: 45.0 ± 13.9; 39 [23-70] | 13 | Immunotherapy | 8-28 months |

**Supplementary Table 7** Studies regarding the treatment of cUGS with ectopic pregnancy or infertility.

| Ectopic pregnancy | | | | | | | | | | |
| --- | --- | --- | --- | --- | --- | --- | --- | --- | --- | --- |
| Ref. | Author | Year of publication | Publishing country | Country of infection | Study type | Patient type | Age | n | Treatment type | Follow-up length |
| [160] | Aminu MB | 2014 | Nigeria | Nigeria | Case report | Endemic area residents | 25 | 1 | Surgery + PZQ |  |
| [75] | Bahrami S | 2006 | USA | East Africa | Case report | Migrants | 20 | 1 | Surgery + PZQ |  |
| [76] | Bugalho A | 1991 | Mozambique | Mozambique | Case series | Endemic area residents | 21; 22; 28; 33 | 4 | Surgery |  |
| [161] | De Muylder X | 1991 | Zimbabwe | Zimbabwe | Prospective cohort | Endemic area residents | unspecified | 2 | Surgery |  |
| [77] | Ekoukou D | 1995 | France | Mali | Case series | Migrants | 29 | 1 | Surgery + PZQ | 3 months |
| [78] | Eogan M | 2002 | Ireland | Nigeria | Case report | Migrants | 31 | 1 | Surgery | 6 weeks |
| [79] | Garba M | 2004 | Niger | Niger | Case report | Endemic area residents | 21 | 1 | Surgery + PZQ | 2 years |
| [80] | Laroche J | 2016 | France | Mali | Case report | Travelers | 26 | 1 | Surgery + PZQ and metothrexate |  |
| [81] | Laxman VV | 2008 | UK | Zambia | Case report | Migrants | 37 | 1 | Surgery + PZQ |  |
| [83] | Odubamowo KH | 2014 | Nigeria | Nigeria | Case report | Endemic area residents | 32 | 1 | Surgery + PZQ | 6 weeks |
| [84] | Okonofua FE | 1990 | Nigeria | Nigeria | Case report | Endemic area residents | 34 | 1 | Surgery + niridazole | 6 weeks |
| [97] | Owusu-Bempah A | 2013 | Ghana | Ghana | Case report | Endemic area residents | 34 | 1 | Surgery | 3 years |
| [85] | Sahu L | 2013 | India | unknown | Case report | Travelers | 25 | 1 | Surgery + PZQ |  |
| [86] | Schneider D | 2000 | South Africa | South Africa | Case report | Endemic area residents | 35 | 1 | Surgery + PZQ |  |
| [87] | Ville Y | 1991 | Gabon | Gabon | Case series | Endemic area residents | 22; 22; 26 | 3 | Surgery |  |
| Female infertility | | | | | | | | | | |
| Ref. | Author | Year of publication | Study country | Country of infection | Study type | Patient type | Age | n | Treatment type | Follow-up length |
| [88] | Bailey SL | 2011 | UK | Malawi | Case series | Travelers | 43 | 1 | PZQ only |  |
| [89] | Balasch J | 1995 | Spain | Nigeria | Case report | Migrants | 26 | 1 | Surgery |  |
| [90] | Darwish AM | 1999 | Egypt | Egypt | Case series | Endemic area residents | 26; 30; 34 | 3 | Surgery |  |
| [91] | Jones KD | 2003 | UK | South Africa | Case report | Travelers | 33 | 1 | PZQ only |  |
| [94] | Krolikowski A | 1995 | South Africa | South Africa | Case report | Endemic area residents | 27 | 1 | Surgery |  |
| [95] | Morice P | 1993 | France | unknown | Case report | Migrants | 33 | 1 | Surgery |  |
| [97] | Owusu-Bempah A | 2013 | Ghana | Ghana | Case report | Endemic area residents | 34 | 1 | Surgery |  |
|  |  |  |  |  |  |  |  | 1 | IVF |  |
| [100] | Schanz A | 2010 | Germany | Nigeria | Case report | Migrants | 30 | 1 | IVF |  |
|  |  |  |  |  |  |  |  | 1 | Surgery |  |
| [102] | Van Den Broucke S | 2020 | Belgium | Mali | Case report | Travelers | 28 | 1 | PZQ only | 5 months |
|  |  |  |  |  |  |  |  | 1 | PZQ only | ongoing |
| Male infertility | | | | | | | | | | |
| Ref. | Author | Year of publication | Study country | Country of infection | Study type | Patient type | Age | n | Treatment type | Follow-up length |
| [103] | Al-Qahtani SM | 2010 | France | Egypt | Case report | Migrants | 31 | 1 | Surgery | 6 months |
| [104] | Kini S | 2009 | UK | Uganda | Case report | Travelers | unspecified | 1 | IVF | 9 months |

**Supplementary Table 8** Case report and case series regarding the treatment of patients requiring surgery or invasive procedures in a not otherwise complicated disease.

| Ref. | Author | Year of publication | Publishing country | Country of infection | Patient type | Sex | Age | Lesion | n | Treatment | Outcome | Follow-up length |
| --- | --- | --- | --- | --- | --- | --- | --- | --- | --- | --- | --- | --- |
| [106] | Al-Saeed O | 2003 | Kuwait | Kuwait | Endemic area residents | m | range: [33-45] | Seminal vesicle mass | 2 | Excision of the mass | Unspecified | – |
| [108] | Azami MA | 2018 | Morocco | Morocco | Endemic area residents | f | 28 | Infected ovarian cyst | 1 | Excision of the cyst, bilateral adnexectomy, appendectomy, ablation of a part of the necrotic epiploon, followed by praziquantel single dose (40 mg/kg) | Unspecified | Ongoing |
| [109] | Badmus TA | 2012 | Nigeria | Nigeria | Endemic area residents | m | 16 | Testicular mass | 1 | Radical orchiectomy, followed by PZQ | Fully recovered | 26 months |
| [88] | Bailey SL | 2011 | UK | Malawi | Travelers | f | 34 | Infected ovarian cyst | 1 | Unilateral adnexiectomy, followed by praziquantel | Unspecified | – |
| [90] | Darwish AM | 1999 | Egypt | Egypt | Endemic area residents | f | 34 | Pelvic pain | 1 | Adhesiolysis and salpingectomy | Unspecified | – |
| [162] | Drew LB | 2018 | Malawi | Malawi | Endemic area residents | f | 20; 35 | Vesicovaginal fistula | 2 | Fistula repair, followed by praziquantel 40 mg/kg | Fully recovered | Unspecified |
| [116] | Efared B | 2018 | Morocco | Morocco | Endemic area residents | f | 51 | Ovarian mass | 1 | Excision of the cyst | Lost to follow-up | – |
| [118] | Fall I | 1992 | Senegal | Senegal | Endemic area residents | m | 9 | Testicular mass | 1 | Orchiectomy, followed by PZQ | Lost to follow-up | – |
| [121] | Kato-Hayashi N | 2013 | Japan | Mali | Travelers | m | 21 | Infection not responsive to PZQ (required biopsy) | 1 | Multiple courses of PZQ | Lesions almost resolved; subjective symptoms disappeared | 15 months |
| [129] | Oguntunde OA | 2020 | Nigeria | Nigeria | Endemic area residents | m | 9 | Testicular mass | 1 | PZQ | Unspecified | Ongoing |
| [132] | Rambau PF | 2011 | Tanzania | Tanzania | Endemic area residents | m | 9 | Testicular swelling and atrophy | 1 | Orchiectomy, followed by PZQ | Unspecified | – |
| [163] | Silva IM | 2005 | Brazil | Mozambique | Travelers | m | median [range]: 29 [26-55] | Infection not responsive to PZQ (required biopsy) | 26 | Treatment with a single dose of praziquantel 40 mg/kg and retreatment with three more doses of praziquantel 40 mg/kg 15 days apart | 17 fully recovered, 4 fully recovered after retreatment, 5 therapeutic failures | 6-42 months |
| [136] | Silva IM | 2008 | Brazil | Mozambique | Travelers | m | unspecified | Infection not responsive to PZQ (required biopsy) | 1 | Seven courses of PZQ 40 mg/kg | Not fully recovered | 62 months |
| [137] | Soans B | 1999 | Australia | Malawi | Travelers | m | 33 | Testicular mass | 1 | Orchiectomy, followed by PZQ | Fully recovered | 4 months |
| [141] | Torricelli M | 1998 | Italy | Morocco | Migrants | m | 14 | Pseudopolyp | 1 | Cystotomy, followed by PZQ | Fully recovered | 1 year |
| [142] | Turkistani I | 2002 | Saudi Arabia | Saudi Arabia | Endemic area residents | f | 37 | Infestation of a paravaginal tumor | 1 | Excision of the tumor, followed by praziquantel 40 mg/kg single dose | Fully recovered | Unspecified |
| [143] | Ze Ondo C | 2013 | Senegal | Senegal | Endemic area residents | m | 6; 38 | Testicular mass | 2 | Orchiectomy, followed by PZQ 40 mg/kg | Fully recovered | 2-4 years |

**Bibliography**

1. Moher D, Shamseer L, Clarke M, Ghersi D, Liberati A, Petticrew M, et al. Preferred reporting items for systematic review and meta-analysis protocols (PRISMA-P) 2015 statement. Syst Rev. 2015;4:1. https://doi.org/10.1186/2046-4053-4-1.

2. Abdel-Wahab MF, Esmat G, Ramzy I, Fouad R, Abdel-Rahman M, Yosery A, et al. Schistosoma haematobium infection in Egyptian schoolchildren: demonstration of both hepatic and urinary tract morbidity by ultrasonography. Trans R Soc Trop Med Hyg. 1992;86(4):406-9. https://doi.org/10.1016/0035-9203(92)90241-4.

3. Abdel-Wahab MF, Ramzy I, Esmat G, el Kafass H, Strickland GT. Ultrasound for detecting Schistosoma haematobium urinary tract complications: comparison with radiographic procedures. J Urol. 1992;148(2 Pt 1):346-50. https://doi.org/10.1016/s0022-5347(17)36590-4.

4. Antwi S, Aboah KE, Sarpong CK. The unacknowledged impact of urinary schistosomiasis in children: 5 cases from Kumasi, Ghana. Ghana Med J. 2014;48(4):228-33. https://doi.org/10.4314/gmj.v48i4.11.

5. Badmos KB, Popoola AA, Buhari MO, Abdulkadir AY. Ureteric schistosomiasis with obstructive uropathy. J Coll Physicians Surg Pak. 2009;19(7):456-8.

6. Bakari AA, Gadam IA, Aliyu S, Suleiman I, Ahidjo AA, Pindiga UH. Use of mitrofanoff and yang-monti techniques as ureteric substitution for severe schistosomal bilateral ureteric stricture: a case report and review of the literature. Niger J Surg. vol 12012. p. 30-3.

7. Bocanegra García C, Pintar Z, Serres X, Mendioroz J, Moreno M, Gallego S, et al. Ultrasound findings and associated factors to morbidity in Schistosoma haematobium infection in a highly endemic setting. Trop Med Int Health. 2018;23(2):221-8. https://doi.org/10.1111/tmi.13020.

8. Brouwer KC, Ndhlovu PD, Wagatsuma Y, Munatsi A, Shiff CJ. Urinary tract pathology attributed to Schistosoma haematobium: does parasite genetics play a role? Am J Trop Med Hyg. 2003;68(4):456-62.

9. Brouwer KC, Ndhlovu PD, Wagatsuma Y, Munatsi A, Shiff CJ. Epidemiological assessment of Schistosoma haematobium-induced kidney and bladder pathology in rural Zimbabwe. Acta Trop. 2003;85(3):339-47. https://doi.org/10.1016/s0001-706x(02)00262-0.

10. Dabo A, Traoré HA, Diakité M, Kouriba B, Camara F, Coulibaly CO, et al. [Echographic morbidity due to Schistosoma haematobium in a peripheral district of Bamako in Mali, Missabougou]. Bull Soc Pathol Exot. 1995;88(1):11-4.

11. Fataar S, Rudwan M, Bassiony H, Satyanath S. CT of genitourinary calcification due to schistosomiasis. Australas Radiol. 1990;34(3):234-7. https://doi.org/10.1111/j.1440-1673.1990.tb02638.x.

12. Garba A, Campagne G, Poda JN, Parent G, Kambire R, Chippaux JP. [Schistosomiasis in the region of Ziga (Burkina Faso) before the construction of a dam]. Bull Soc Pathol Exot. 1999;92(3):195-7.

13. Ibrahim AI, Patil KP, el Tahir MI, Shetty SD, Anandan N. Bilharzial vesicoureteric reflux and bladder neck stenosis: fact or fiction? Br J Urol. 1991;68(6):582-5. https://doi.org/10.1111/j.1464-410x.1991.tb15419.x.

14. Kazmi Z, Ashfaq MA, Umer D, Idrees R, Ather MH. Snail Fever of the Bladder in a Non-endemic Area. J Coll Physicians Surg Pak. vol 8. Pakistan2020. p. 874-6.

15. Lorca J, Hevia V, Diez Nicolás V, González A, Sánchez Guerrero C, Burgos Revilla FJ. Minimmally invasive resolution of a left ureteral stenosis after Schistosoma haematobium infection. Urol Case Rep. 2019. p. 100889.

16. Mandong BM, Madaki AJ. Missed diagnosis of schistosomiasis leading to unnecessary surgical procedures in Jos University Teaching Hospital. Trop Doct. 2005;35(2):96-7. https://doi.org/10.1258/0049475054037011.

17. Olajide AO, Olajide FO, Aremu AA, Komolafe AO. Ureteric obstruction secondary to schistosomiasis 2 years after praziquantel therapy: a case report. Pan Afr Med J. 2012;12:32.

18. Oranusi CK, Nwofor A, Onyiaorah IV, Ukah CO. Schistosomal stricture of the ureter-diagnostic dilemma. Niger J Clin Pract. 2011;14(4):495-8. https://doi.org/10.4103/1119-3077.91765.

19. Pal PO, Smith RD, Allen S, Ratynska M, Edwards S, Gothard P, et al. Schistosomiasis-A Disobedient Ureter, a Disobedient Diagnosis. J Endourol Case Rep. vol 12017. p. 114-8.

20. Pallangyo P, Bhalia S, Simelane NN, Lyimo F, Swai HJ, Mkojera ZS, et al. Massive Bilateral Hydroureteronephrosis and End-Stage Renal Disease Ensuing From Chronic Schistosomiasis: A Case Report. J Investig Med High Impact Case Rep. 2020;8:2324709620910912. https://doi.org/10.1177/2324709620910912.

21. Pieras Ayala E, Salvador J, Vicente J. [Bilharziasis, clinical course of the disease: acute and chronic phase. Two clinical cases]. Arch Esp Urol. 2000;53(9):834-9.

22. Pollock GR, Meiklejohn KM, Zeng J, Chipollini J. Robotic Cystoprostatectomy With Intracorporeal Ileal Conduit Diversion in a Patient With Chronic Schistosomiasis. Urology. 2020;141:e8-e9. https://doi.org/10.1016/j.urology.2020.04.052.

23. Rasendramino MH, Rajaona HR, Ramarokoto CE, Ravaoalimalala VE, Leutscher P, Cordonnier D, et al. [Prevalence of uro-nephrologic complications of urinary bilharziasis in hyperendemic focus in Madagascar]. Nephrologie. 1998;19(6):341-5.

24. Remppis J, Verheyden A, Bustinduy AL, Heller T, García-Tardón N, Manouana GP, et al. Focused Assessment with Sonography for Urinary Schistosomiasis (FASUS)-pilot evaluation of a simple point-of-care ultrasound protocol and short training program for detecting urinary tract morbidity in highly endemic settings. Trans R Soc Trop Med Hyg. 2020;114(1):38-48. https://doi.org/10.1093/trstmh/trz101.

25. Richter J, Wagatsuma Y, Aryeetey M, Feldmeier H. Sonographic screening for urinary tract abnormalities in patients with Schistosoma haematobium infection: pitfalls in examining pregnant women. Bull World Health Organ. 1996;74(2):217-21.

26. Salah MA. Ultrasonography of urinary tract lesions caused by bilharziasis in Yemeni patients. BJU Int. 2000;86(7):790-3. https://doi.org/10.1046/j.1464-410x.2000.00921.x.

27. Salas-Coronas J, Vázquez-Villegas J, Villarejo-Ordóñez A, Sánchez-Sánchez JC, Espada-Chavarría J, Soriano-Pérez MJ, et al. [Radiological findings in patients with imported schistosomiasis]. Enferm Infecc Microbiol Clin. 2013;31(4):205-9. https://doi.org/10.1016/j.eimc.2012.04.003.

28. Salas-Coronas J, Vázquez-Villegas J, Lozano-Serrano AB, Soriano-Pérez MJ, Cabeza-Barrera I, Cabezas-Fernández MT, et al. Severe complications of imported schistosomiasis, Spain: A retrospective observational study. Travel Med Infect Dis. 2020;35:101508. https://doi.org/10.1016/j.tmaid.2019.101508.

29. Serieye J, Boisier P, Ravaoalimalala VE, Ramarokoto CE, Leutscher P, Esterre P, et al. Schistosoma haematobium infection in western Madagascar: morbidity determined by ultrasonography. Trans R Soc Trop Med Hyg. 1996;90(4):398-401. https://doi.org/10.1016/s0035-9203(96)90521-0.

30. Srougi V, Gallucci FP, Mattedi RL, Srougi M. Carcinosarcoma of the bladder following local schistosomiasis infection. BMJ Case Rep. 2017;2017. https://doi.org/10.1136/bcr-2016-218642.

31. Vancauwenberghe T, Oyaert M, Termote JL, Mulkens T, Bellinck P. Ureteral obstruction caused by schistosomiasis. Jbr-btr. 2013;96(5):292-4. https://doi.org/10.5334/jbr-btr.412.

32. Vester U, Kardorff R, Traoré M, Traoré HA, Fongoro S, Juchem C, et al. Urinary tract morbidity due to Schistosoma haematobium infection in Mali. Kidney Int. 1997;52(2):478-81. https://doi.org/10.1038/ki.1997.356.

33. Abdel Mohsen MA, Hassan AA, El-Sewedy SM, Aboul-Azm T, Magagnotti C, Fanelli R, et al. Biomonitoring of n-nitroso compounds, nitrite and nitrate in the urine of Egyptian bladder cancer patients with or without Schistosoma haematobium infection. Int J Cancer. 1999;82(6):789-94. https://doi.org/10.1002/(sici)1097-0215(19990909)82:6<789::aid-ijc3>3.0.co;2-c.

34. Ahmed NS, Mahmoud SF, Mohamed ER, Khalifa RM. HISTOPATHOLOGICAL ANALYSIS OF SCHISTOSOMA HAEMATOBIUM METAPLASIA OF THE URINARY BLADDER. J Egypt Soc Parasitol. 2017;47(1):211-8.

35. Ahmed SA, Gad MZ. Diagnostic value of serum lactate dehydrogenase isoenzyme and amino acid patterns in several schistosomal and non-schistosomal disorders as compared to other biochemical parameters. Dis Markers. 1996;13(1):19-29. https://doi.org/10.1155/1996/214869.

36. Akinwale OP, Oliveira GC, Ajayi MB, Akande DO, Oyebadejo S, Okereke KC. Squamous cell abnormalities in exfoliated cells from the urine of Schistosoma haematobium-infected adults in a rural fishing community in Nigeria. World Health Popul. 2008;10(1):18-22. https://doi.org/10.12927/whp.2008.19581.

37. Al-Samawi AS, Aulaqi SM. Urinary bladder cancer in yemen. Oman Med J. 2013;28(5):337-40. https://doi.org/10.5001/omj.2013.97.

38. Alvarez Kindelan J, Alameda Aragoneses V, Carmona Campos E, Anglada Curado F, Prieto Castro R, Regueiro López JC, et al. [Bilharziasis and bladder cancer. A case report]. Actas Urol Esp. 1999;23(1):60-3.

39. Amin HAA, Kobaisi MH, Samir RM. Schistosomiasis and Bladder Cancer in Egypt: Truths and Myths. Open Access Maced J Med Sci. 2019;7(23):4023-9. https://doi.org/10.3889/oamjms.2019.857.

40. Bedwani R, Renganathan E, El Kwhsky F, Braga C, Abu Seif HH, Abul Azm T, et al. Schistosomiasis and the risk of bladder cancer in Alexandria, Egypt. Br J Cancer. 1998;77(7):1186-9. https://doi.org/10.1038/bjc.1998.197.

41. Darré T, Kpatcha M, Tchaou M, Amégbor K, Sonhaye L, N'Timon B, et al. [Histological aspect of urinary schistosomiasis in Togo: results of a cohort of 192 cases]. Bull Soc Pathol Exot. 2015;108(2):124-5. https://doi.org/10.1007/s13149-015-0427-4.

42. Eissa S, Matboli M, Awad N, Kotb Y. Identification and validation of a novel autophagy gene expression signature for human bladder cancer patients. Tumour Biol. 2017;39(4):1010428317698360. https://doi.org/10.1177/1010428317698360.

43. Eissa S, Matboli M, Essawy NO, Kotb YM. Integrative functional genetic-epigenetic approach for selecting genes as urine biomarkers for bladder cancer diagnosis. Tumour Biol. 2015;36(12):9545-52. https://doi.org/10.1007/s13277-015-3722-6.

44. Eissa S, Ali-Labib R, Swellam M, Bassiony M, Tash F, El-Zayat TM. Noninvasive diagnosis of bladder cancer by detection of matrix metalloproteinases (MMP-2 and MMP-9) and their inhibitor (TIMP-2) in urine. Eur Urol. 2007;52(5):1388-96. https://doi.org/10.1016/j.eururo.2007.04.006.

45. Eissa S, Badr S, Barakat M, Zaghloul AS, Mohanad M. The diagnostic efficacy of urinary survivin and hyaluronidase mRNA as urine markers in patients with bladder cancer. Clin Lab. 2013;59(7-8):893-900. https://doi.org/10.7754/clin.lab.2012.120623.

46. Eissa S, Habib H, Ali E, Kotb Y. Evaluation of urinary miRNA-96 as a potential biomarker for bladder cancer diagnosis. Med Oncol. 2015;32(1):413. https://doi.org/10.1007/s12032-014-0413-x.

47. Eissa S, Kassim SK, Labib RA, El-Khouly IM, Ghaffer TM, Sadek M, et al. Detection of bladder carcinoma by combined testing of urine for hyaluronidase and cytokeratin 20 RNAs. Cancer. 2005;103(7):1356-62. https://doi.org/10.1002/cncr.20902.

48. Eissa S, Kenawy G, Swellam M, El-Fadle AA, Abd El-Aal AA, El-Ahmady O. Comparison of cytokeratin 20 RNA and angiogenin in voided urine samples as diagnostic tools for bladder carcinoma. Clin Biochem. 2004;37(9):803-10. https://doi.org/10.1016/j.clinbiochem.2004.05.027.

49. Eissa S, Labib RA, Mourad MS, Kamel K, El-Ahmady O. Comparison of telomerase activity and matrix metalloproteinase-9 in voided urine and bladder wash samples as a useful diagnostic tool for bladder cancer. Eur Urol. 2003;44(6):687-94. https://doi.org/10.1016/s0302-2838(03)00417-2.

50. Eissa S, Matboli M, Essawy NO, Shehta M, Kotb YM. Rapid detection of urinary long non-coding RNA urothelial carcinoma associated one using a PCR-free nanoparticle-based assay. Biomarkers. 2015;20(3):212-7. https://doi.org/10.3109/1354750x.2015.1062918.

51. Eissa S, Matboli M, Hegazy MG, Kotb YM, Essawy NO. Evaluation of urinary microRNA panel in bladder cancer diagnosis: relation to bilharziasis. Transl Res. 2015;165(6):731-9. https://doi.org/10.1016/j.trsl.2014.12.008.

52. Eissa S, Matboli M, Mansour A, Mohamed S, Awad N, Kotb YM. Evaluation of urinary HURP mRNA as a marker for detection of bladder cancer: relation to bilharziasis. Med Oncol. 2014;31(2):804. https://doi.org/10.1007/s12032-013-0804-4.

53. Eissa S, Motawi T, Badr S, Zaghlool A, Maher A. Evaluation of urinary human telomerase reverse transcriptase mRNA and scatter factor protein as urine markers for diagnosis of bladder cancer. Clin Lab. 2013;59(3-4):317-23. https://doi.org/10.7754/clin.lab.2012.120507.

54. Eissa S, Swellam M, Ali-Labib R, Mansour A, El-Malt O, Tash FM. Detection of telomerase in urine by 3 methods: evaluation of diagnostic accuracy for bladder cancer. J Urol. 2007;178(3 Pt 1):1068-72. https://doi.org/10.1016/j.juro.2007.05.006.

55. Eissa S, Swellam M, Sadek M, Mourad MS, El Ahmady O, Khalifa A. Comparative evaluation of the nuclear matrix protein, fibronectin, urinary bladder cancer antigen and voided urine cytology in the detection of bladder tumors. J Urol. 2002;168(2):465-9.

56. Eissa S, Zohny SF, Swellam M, Mahmoud MH, El-Zayat TM, Salem AM. Comparison of CD44 and cytokeratin 20 mRNA in voided urine samples as diagnostic tools for bladder cancer. Clin Biochem. 2008;41(16-17):1335-41. https://doi.org/10.1016/j.clinbiochem.2008.08.085.

57. el-Ahmady O, Halim AB, el-Din AG. The clinical value of CYFRA21-1 in bladder cancer patients: Egyptian experience. Anticancer Res. 1999;19(4a):2603-8.

58. El-Sharkawi F, El Sabah M, Hassan Z, Khaled H. The biochemical value of urinary metalloproteinases 3 and 9 in diagnosis and prognosis of bladder cancer in Egypt. J Biomed Sci. 2014;21(1):72. https://doi.org/10.1186/s12929-014-0072-4.

59. Gaber DA, Wassef RM, El-Ayat WM, El-Moazen MI, Montasser KA, Swar SA, et al. Role of a schistosoma haematobium specific microRNA as a predictive and prognostic tool for bilharzial bladder cancer in Egypt. Sci Rep. 2020;10(1):18844. https://doi.org/10.1038/s41598-020-74807-1.

60. Gaye AM, Doh K, Thiam I, Bentefouet L, Woto-Gaye G. [Schistosomiasis and cancer: A fortuitous association or relationships cause and effect]. Bull Cancer. 2016;103(9):806-7. https://doi.org/10.1016/j.bulcan.2016.07.002.

61. Groeneveld AE, Marszalek WW, Heyns CF. Bladder cancer in various population groups in the greater Durban area of KwaZulu-Natal, South Africa. Br J Urol. 1996;78(2):205-8. https://doi.org/10.1046/j.1464-410x.1996.09310.x.

62. Ketabchi A, Moshtaghi-Kashanian G. Urinary schistosomiasis with simultaneous bladder squamous cell carcinoma and transitional cell carcinoma. Iran J Parasitol. vol 32012. p. 96-8.

63. Khaled HM, Abdel-Salam I, Abdel-Gawad M, Metwally A, El-Demerdash S, El-Didi M, et al. Evaluation of the BTA tests for the detection of bilharzial related bladder cancer: the Cairo experience. Eur Urol. 2001;39(1):91-4. https://doi.org/10.1159/000052418.

64. Lodhia J, Mremi A, Pyuza JJ, Bartholomeo N, Herman AM. Schistosomiasis and cancer: Experience from a zonal hospital in Tanzania and opportunities for prevention. J Surg Case Rep. vol 5: Published by Oxford University Press and JSCR Publishing Ltd. All rights reserved. © The Author(s) 2020.; 2020. p. rjaa144.

65. Marbjerg LH, Øvrehus AL, Johansen IS. Schistosomiasis-induced squamous cell bladder carcinoma in an HIV-infected patient. Int J Infect Dis. 2015;40:113-5. https://doi.org/10.1016/j.ijid.2015.10.004.

66. Martin JW, Vernez SL, Lotan Y, Abdelhalim A, Dutta R, Shokeir A, et al. Pathological characteristics and prognostic indicators of different histopathological types of urinary bladder cancer following radical cystectomy in a large single-center Egyptian cohort. World J Urol. 2018;36(11):1835-43. https://doi.org/10.1007/s00345-018-2331-6.

67. Mina SN, Antonios SN. LYMPHOEPITHELIOMA-LIKE CARCINOMA OF THE URINARY BLADDER ASSOCIATED WITH SCHISTOSOMIASIS: A CASE REPORT AND REVIEW OF LITERATURE. J Egypt Soc Parasitol. 2015;45(2):385-8. https://doi.org/10.12816/0017583.

68. Mohammed MA, Seleim MF, Abdalla MS, Sharada HM, Abdel Wahab AH. Urinary high molecular weight matrix metalloproteinases as non-invasive biomarker for detection of bladder cancer. BMC Urol. 2013;13:25. https://doi.org/10.1186/1471-2490-13-25.

69. Mourad WA, Khalil S, Radwi A, Peracha A, Ezzat A. Primary T-cell lymphoma of the urinary bladder. Am J Surg Pathol. 1998;22(3):373-7. https://doi.org/10.1097/00000478-199803000-00014.

70. Mungadi IA, Malami SA. Urinary bladder cancer and schistosomiasis in North-Western Nigeria. West Afr J Med. 2007;26(3):226-9. https://doi.org/10.4314/wajm.v26i3.28315.

71. Saied GM, El-Metenawy WH, Elwan MS, Dessouki NR. Urine carcinoembryonic antigen levels are more useful than serum levels for early detection of Bilharzial and non-Bilharzial urinary bladder carcinoma: observations of 43 Egyptian cases. World J Surg Oncol. 2007;5:4. https://doi.org/10.1186/1477-7819-5-4.

72. Santos J, Chaves J, Araújo H, Vale N, Costa JM, Brindley PJ, et al. Comparison of findings using ultrasonography and cystoscopy in urogenital schistosomiasis in a public health centre in rural Angola. S Afr Med J. 2015;105(4):312-5. https://doi.org/10.7196/samj.8564.

73. Vieira P, Miranda HP, Cerqueira M, Delgado Mde L, Coelho H, Antunes D, et al. Latent schistosomiasis in Portuguese soldiers. Mil Med. 2007;172(2):144-6. https://doi.org/10.7205/milmed.172.2.144.

74. Yang H, Yang K, Khafagi A, Tang Y, Carey TE, Opipari AW, et al. Sensitive detection of human papillomavirus in cervical, head/neck, and schistosomiasis-associated bladder malignancies. Proc Natl Acad Sci U S A. 2005;102(21):7683-8. https://doi.org/10.1073/pnas.0406904102.

75. Bahrami S, Alatassi H, Slone SP, O'Connor DM. Tubal gestation and schistosomiasis: a case report. J Reprod Med. 2006;51(7):595-8.

76. Bugalho A, Strolego F, Benussi G, Pregazzi R, Osman N. [Schistosomiasis: possible cause of ectopic pregnancy. Four clinical cases]. Minerva Ginecol. 1991;43(12):577-9.

77. Ekoukou D, Luzolo-Lukanu A, Mulard C, Bazin C, Ng Wing Tin L. [Peritoneal and tubal Schistosoma haematobium bilharziasis. Two case reports]. J Gynecol Obstet Biol Reprod (Paris). 1995;24(8):819-24.

78. Eogan M, O'Malley A, Flavin R, Gillan J, McKenna P, Coulter-Smith S. Ectopic pregnancy associated with tubal schistosomiasis. Ir Med J. 2002;95(8):250.

79. Garba M, Almoustapha T, Garba A, Nouhou H. [Extra uterine pregnancy associated with a tubal schistosomiasis due to Schistosoma haematobium. A case report from Niger]. Bull Soc Pathol Exot. 2004;97(1):41-2.

80. Laroche J, Mottet N, Malincenco M, Gay C, Royer PY, Riethmuller D. [Successive ectopic pregnancies associated with tubal shistosomiasis in a French traveler]. Pan Afr Med J. 2016;23:18. https://doi.org/10.11604/pamj.2016.23.18.8845.

81. Laxman VV, Adamson B, Mahmood T. Recurrent ectopic pregnancy due to Schistosoma hematobium. J Obstet Gynaecol. 2008;28(4):461-2. https://doi.org/10.1080/01443610802164896.

82. Nouhou H, Sève B, Idi N, Moussa F. [Schistosomiasis of the female genital tract: anatomoclinical and histopathological aspects. Apropos of 26 cases]. Bull Soc Pathol Exot. 1998;91(3):221-3.

83. Odubamowo KH, Akinpelu OM, Lawal OO, Okolo CA, Odukogbe AA, Adekunle AO. Bilateral tubal gestation associated with schistosomiasis in an african woman. Case Rep Obstet Gynecol. 2014;2014:674514. https://doi.org/10.1155/2014/674514.

84. Okonofua FE, Ojo OS, Odunsi OA, Odesanmi WO. Ectopic pregnancy associated with tubal schistosomiasis in a Nigerian woman. Int J Gynaecol Obstet. 1990;32(3):281-4. https://doi.org/10.1016/0020-7292(90)90359-s.

85. Sahu L, Tempe A, Singh S, Khurana N. Ruptured ectopic pregnancy associated with tubal schistosomiasis. J Postgrad Med. 2013;59(4):315-7. https://doi.org/10.4103/0022-3859.123166.

86. Schneider D, Steyn DW. Genital schistosomiasis presenting as suspected ectopic pregnancy in the Western Cape. S Afr Med J. 2000;90(6):609.

87. Ville Y, Leruez M, Picaud A, Walter P, Fernandez H. Tubal schistosomiasis as a cause of ectopic pregnancy in endemic areas?; a report of three cases. Eur J Obstet Gynecol Reprod Biol. 1991;42(1):77-9. https://doi.org/10.1016/0028-2243(91)90164-g.

88. Bailey SL, Price J, Llewelyn M. Fluke infertility: the late cost of a quick swim. J Travel Med. 2011;18(1):61-2. https://doi.org/10.1111/j.1708-8305.2010.00476.x.

89. Balasch J, Martínez-Román S, Creus M, Campo E, Fortuny A, Vanrell JA. Schistosomiasis: an unusual cause of tubal infertility. Hum Reprod. 1995;10(7):1725-7. https://doi.org/10.1093/oxfordjournals.humrep.a136163.

90. Darwish AM. Laparoscopic evidence of upper genital schistosomiasis. J Obstet Gynaecol. 1999;19(2):122-4. https://doi.org/10.1080/01443619965381.

91. Jones KD, Okaro EO, Sutton C. The laparoscopic appearance of Schistosomiasis may be mistaken for "non-pigmented" endometriosis. Eur J Obstet Gynecol Reprod Biol. 2003;106(2):227-9. https://doi.org/10.1016/s0301-2115(02)00220-8.

92. Kjetland EF, Poggensee G, Helling-Giese G, Richter J, Sjaastad A, Chitsulo L, et al. Female genital schistosomiasis due to Schistosoma haematobium. Clinical and parasitological findings in women in rural Malawi. Acta Trop. 1996;62(4):239-55. https://doi.org/10.1016/s0001-706x(96)00026-5.

93. Kjetland EF, Kurewa EN, Mduluza T, Midzi N, Gomo E, Friis H, et al. The first community-based report on the effect of genital Schistosoma haematobium infection on female fertility. Fertil Steril. 2010;94(4):1551-3. https://doi.org/10.1016/j.fertnstert.2009.12.050.

94. Krolikowski A, Janowski K, Larsen JV. Asherman syndrome caused by schistosomiasis. Obstet Gynecol. 1995;85(5 Pt 2):898-9. https://doi.org/10.1016/0029-7844(94)00371-j.

95. Morice P, Gadonneix P, Van den Akker M, Antoine M, Villet R. [Tubal bilharziasis]. J Gynecol Obstet Biol Reprod (Paris). 1993;22(8):848-50.

96. Nayama M, Garba A, Boulama-Jackou ML, Touré A, Idi N, Garba M, et al. [Uro-genital schistosomiasis with S. haematobium and infertility in Niger. Prospective study of 109 cases]. Mali Med. 2007;22(3):15-21.

97. Owusu-Bempah A, Odoi AT, Dassah ET. Genital schistosomiasis leading to ectopic pregnancy and subfertility: a case for parasitic evaluation of gynaecologic patients in schistosomiasis endemic areas. Case Rep Obstet Gynecol. 2013;2013:634264. https://doi.org/10.1155/2013/634264.

98. Richter J, Poggensee G, Helling-Giese G, Kjetland E, Chitsulo L, Koumenda N, et al. Transabdominal ultrasound for the diagnosis of Schistosoma haematobium infection of the upper female genital tract: a preliminary report. Trans R Soc Trop Med Hyg. 1995;89(5):500-1. https://doi.org/10.1016/0035-9203(95)90084-5.

99. Santos J, Gouveia MJ, Vale N, Delgado Mde L, Gonçalves A, da Silva JM, et al. Urinary estrogen metabolites and self-reported infertility in women infected with Schistosoma haematobium. PLoS One. 2014;9(5):e96774. https://doi.org/10.1371/journal.pone.0096774.

100. Schanz A, Richter J, Beyer I, Baldus SE, Hess AP, Kruessel JS. Genital schistosomiasis as a cause of female sterility and acute abdomen. Fertil Steril. 2010;93(6):2075.e7-9. https://doi.org/10.1016/j.fertnstert.2009.05.043.

101. Swai B, Poggensee G, Mtweve S, Krantz I. Female genital schistosomiasis as an evidence of a neglected cause for reproductive ill-health: a retrospective histopathological study from Tanzania. BMC Infect Dis. 2006;6:134. https://doi.org/10.1186/1471-2334-6-134.

102. Van Den Broucke S, Potters I, Van Esbroeck M, Cnops L, Siozopoulou V, Hammoud C, et al. A Woman With Chronic Lower Abdominal Pain, Vaginal Discharge, and Infertility After a Stay in Mali. Open Forum Infect Dis. vol 52020. p. ofaa133.

103. Al-Qahtani SM, Droupy SJ. Testicular schistosomiasis. Saudi Med J. 2010;31(3):325-7.

104. Kini S, Dayoub N, Raja A, Pickering S, Thong J. Schistosomiasis-induced male infertility. BMJ Case Rep. 2009;2009. https://doi.org/10.1136/bcr.01.2009.1481.

105. Alvarez Maestro M, Rios Gonzalez E, Dominguez Garcia P, Vallejo Herrador J, Diez Rodriguez J, Martinez-Piñeiro L. Bladder schistosomiasis: case report and bibliographic review. Arch Esp Urol. 2010;63(7):554-8. https://doi.org/10.4321/s0004-06142010000700013.

106. Al-Saeed O, Sheikh M, Kehinde EO, Makar R. Seminal vesicle masses detected incidentally during transrectal sonographic examination of the prostate. J Clin Ultrasound. 2003;31(4):201-6. https://doi.org/10.1002/jcu.10158.

107. Aytaç B, Sehıtoğlu I. A rare parasitic infection in Turkey: schistosomiasis. Case report. Turk Patoloji Derg. 2012;28(2):175-7. https://doi.org/10.5146/tjpath.2012.01120.

108. Azami MA, Elalami I, Siati A, Lamalmi N. An unusual presentation of ovarian dermoid cyst: a case report and review of literature. Obstet Gynecol Sci. 2018;61(4):529-32. https://doi.org/10.5468/ogs.2018.61.4.529.

109. Badmus TA, Takure AO, Osasan SA, Olajide AO, Sabageh DO. Testicular schistosomiasis: a case report. Niger Postgrad Med J. 2012;19(1):50-1.

110. Ballesta Martínez B, Rodríguez Talavera J, Amador Robayna A, Carrión Valencia A, Orribo Morales N, García García L, et al. Parasitic Hematuria: Six Cases in a Row in a Single Centre in Spain. Urol Int. vol 3. Switzerland: © 2017 S. Karger AG, Basel.; 2019. p. 360-3.

111. Carrión López P, Pastor Navarro H, Martínez Ruiz J, Martínez Sanchiz C, Donate Moreno MJ, Segura Martín M, et al. Cystoscopy in bladder bilharziasis. Arch Esp Urol. 2010;63(1):85-6.

112. Chahdi H, Damiri A, El Ochi MR, Allaoui M, Al Bouzidi A, Oukabli M. Urinary schistosomiasis: report of case diagnosed in bladder biopsy. BMC Clin Pathol. 2018. p. 13.

113. De NV, La T, Minh PN, Dao PTB, Duyet LV. Detection of four patients who were infected by Schistosoma haematobium in Vietnam. Infect Drug Resist. 2019;12:439-45. https://doi.org/10.2147/idr.s179746.

114. Dessyn JF, Duquenne S, Hoarau G. Incidental pseudolymphomatous bladder inflammatory polyp revealing urinary schistosomiasis. Int J Infect Dis. 2016;53:39-40. https://doi.org/10.1016/j.ijid.2016.10.022.

115. Dzeing-Ella A, Mechaï F, Consigny PH, Zerat L, Viard JP, Lecuit M, et al. Cervical schistosomiasis as a risk factor of cervical uterine dysplasia in a traveler. Am J Trop Med Hyg. 2009;81(4):549-50. https://doi.org/10.4269/ajtmh.2009.08-0498.

116. Efared B, Sidibé IS, Erregad F, Hammas N, Chbani L, Fatemi HE. Schistosomiasis mimicking ovarian neoplasm. Trop Doct. 2018;48(3):238-40. https://doi.org/10.1177/0049475518770574.

117. Fabiano M, Califano A, Chiancone F, D'Antonio A, Maiorino F, Simeone D, et al. Bladder schistosomiasis in Italy: A case report. Urologia. 2020;87(4):191-3. https://doi.org/10.1177/0391560320910647.

118. Fall I, N'Doye M, Wandaogo A, Sankale AA, Diop A. [A case report of epididymo-testicular bilharziasis in a child]. Ann Urol (Paris). 1992;26(6-7):360-1.

119. Haghighi L, Akbaribazm M, Arab-Mazar Z, Rahimi M. Vesical schistosomiasis and squamous cell carcinoma associated with schistosoma haematobium: A re-emerging neglected tropical disease in Tehran, Iran. Urol Case Rep. © 2020 Published by Elsevier Inc.; 2020. p. 101140.

120. Hosny K, Luk A. Urinary schistosomiasis presented as bladder malignancy with pulmonary metastases: a case report. Ann R Coll Surg Engl. 2018;100(6):e145-e6. https://doi.org/10.1308/rcsann.2018.0072.

121. Kato-Hayashi N, Yasuda M, Yuasa J, Isaka S, Haruki K, Ohmae H, et al. Use of cell-free circulating schistosome DNA in serum, urine, semen, and saliva to monitor a case of refractory imported schistosomiasis hematobia. J Clin Microbiol. 2013;51(10):3435-8. https://doi.org/10.1128/jcm.01219-13.

122. Kameh D, Smith A, Brock MS, Ndubisi B, Masood S. Female genital schistosomiasis: case report and review of the literature. South Med J. 2004;97(5):525-7. https://doi.org/10.1097/00007611-200405000-00022.

123. Kohno M, Kuwatsuru R, Suzuki K, Nishii N, Hayano T, Mitsuhashi N, et al. Imaging findings from a case of bilharziasis in a patient with gross hematuria of several years' duration. Radiat Med. 2008;26(9):553-6. https://doi.org/10.1007/s11604-008-0274-3.

124. Labairu Huerta L, Cuesto Alcalá JA, Napal Lecumberri S, Gómez Dorronsoro M, Pascual Piédrola JI. [Bilharziasis. Case report]. Arch Esp Urol. 2007;60(7):795-9. https://doi.org/10.4321/s0004-06142007000700010.

125. Lee Y, Song HB, Jung BK, Choe G, Choi MH. Case Report of Urinary Schistosomiasis in a Returned Traveler in Korea. Korean J Parasitol. 2020;58(1):51-5. https://doi.org/10.3347/kjp.2020.58.1.51.

126. López López AI, Cao Avellaneda E, Prieto González A, Ferri Níguez B, Maluff Torres A, Pérez Albacete M. [Schistosomiasis: not an uncommon parasitosis in Europe]. Actas Urol Esp. 2007;31(8):915-8. https://doi.org/10.1016/s0210-4806(07)73747-6.

127. Mascarenhas A, Castro I. A rare case of hematuria. Einstein (Sao Paulo). 2011;9(1):81-3. https://doi.org/10.1590/s1679-45082011rc1946.

128. Neal PM. Schistosomiasis--an unusual cause of ureteral obstruction: a case history and perspective. Clin Med Res. 2004;2(4):216-27. https://doi.org/10.3121/cmr.2.4.216.

129. Oguntunde OA, Ikhisemojie S, Sonusi SE, Oyebode A, Abdulkareem B, Banjo AA. Testicular schistosomiasis mimicking hydrocele in a child: a case report. Pan Afr Med J. © Olubanji Ajibola Oguntunde et al.; 2020. p. 56.

130. Pedalino M, Vercesi E, Manini C, Piras D, Di Primio OG, Vella R, et al. [A case of chronic schistosomiasis four years after infestation]. Urologia. 2010;77 Suppl 17:38-41.

131. Pinto SZ, Friedman R, Van Den Berg EJ. A case of paediatric bladder bilharzioma in Johannesburg, South Africa. Clin Case Rep. vol 10: © 2019 The Authors. Clinical Case Reports published by John Wiley & Sons Ltd.; 2019. p. 1890-4.

132. Rambau PF, Chandika A, Chalya PL, Jackson K. Scrotal Swelling and Testicular Atrophy due to Schistosomiasis in a 9-Year-Old Boy: A Case Report. Case Rep Infect Dis. 2011;2011:787961. https://doi.org/10.1155/2011/787961.

133. Samuel MI, Taylor C. A case of female urogenital schistosomiasis presenting as viral warts. Int J STD AIDS. 2015;26(8):599-601. https://doi.org/10.1177/0956462414544723.

134. Scarlata F, Giordano S, Romano A, Marasa L, Lipani G, Infurnari L, et al. [Urinary schistosomiasis: remarks on a case]. Infez Med. 2005;13(4):259-64.

135. Silva IM, Thiengo R, Conceição MJ, Rey L, Pereira Filho E, Ribeiro PC. Cystoscopy in the diagnosis and follow-up of urinary schistosomiasis in Brazilian soldiers returning from Mozambique, Africa. Rev Inst Med Trop Sao Paulo. 2006;48(1):39-42. https://doi.org/10.1590/s0036-46652006000100008.

136. Silva IM, Pereira Filho E, Thiengo R, Ribeiro PC, Conceição MJ, Panasco M, et al. Schistosomiasis haematobia: histopathological course determined by cystoscopy in a patient in whom praziquantel treatment failed. Rev Inst Med Trop Sao Paulo. 2008;50(6):343-6. https://doi.org/10.1590/s0036-46652008000600006.

137. Soans B, Abel C. Ultrasound appearance of schistosomiasis of the testis. Australas Radiol. 1999;43(3):385-7. https://doi.org/10.1046/j.1440-1673.1999.433685.x.

138. Sultana SR, Byrne DJ, McCullough JB. The value of a travel history in urology. Scott Med J. 1995;40(3):83. https://doi.org/10.1177/003693309504000308.

139. Tan WP, Hwang T, Park JW, Elterman L. Schistosoma haematobium: A Delayed Cause of Hematuria. Urology. 2017;107:e7-e8. https://doi.org/10.1016/j.urology.2017.06.021.

140. Tilli M, Gobbi F, Rinaldi F, Testa J, Caligaris S, Magro P, et al. The diagnosis and treatment of urogenital schistosomiasis in Italy in a retrospective cohort of immigrants from Sub-Saharan Africa. Infection. 2019;47(3):447-59. https://doi.org/10.1007/s15010-019-01270-0.

141. Torricelli M, Cerri M, Leva E, Magro P, Roma G, Runza L, et al. [An unusual case of macroscopic hematuria in pediatric age]. Pediatr Med Chir. 1998;20(1):81-3.

142. Turkistani I, Ghourab S, Al-Rikabi A, Al-Sheikh AE, Al-Orainy I. Large paravaginal solitary fibrous tumor with secondary schistosoma hematobium infestation. Acta Obstet Gynecol Scand. 2002;81(1):88-90. https://doi.org/10.1034/j.1600-0412.2002.810116.x.

143. Ze Ondo C, Sarr A, Sow Y, Thiam I, Fall B, Sow D, et al. [Testicular bilharzioma by Schistosomia haematobium: about two cases]. Prog Urol. 2014;24(1):67-9. https://doi.org/10.1016/j.purol.2013.04.016.

144. Zepeda CM, Coffey KH. Schistosoma haematobium Infection That Mimics Bladder Cancer in a 66-Year-Old Ethnic Egyptian Man. Lab Med. 2015;46(4):338-42. https://doi.org/10.1309/lm96ejppbyadiovc.

145. Garba A, Campagne G, Tassie JM, Barkire A, Vera C, Sellin B, et al. [Long-term impact of a mass treatment by praziquantel on morbidity due to Schistosoma haematobium in two hyperendemic villages of Niger]. Bull Soc Pathol Exot. 2004;97(1):7-11.

146. Hatz C, Mayombana C, de Savigny D, MacPherson CN, Koella JC, Degrémont A, et al. Ultrasound scanning for detecting morbidity due to Schistosoma haematobium and its resolution following treatment with different doses of praziquantel. Trans R Soc Trop Med Hyg. 1990;84(1):84-8. https://doi.org/10.1016/0035-9203(90)90392-r.

147. Kardorff R, Traoré M, Doehring-Schwerdtfeger E, Vester U, Ehrich JH. Ultrasonography of ureteric abnormalities induced by Schistosoma haematobium infection before and after praziquantel treatment. Br J Urol. 1994;74(6):703-9. https://doi.org/10.1111/j.1464-410x.1994.tb07110.x.

148. King CH, Lombardi G, Lombardi C, Greenblatt R, Hodder S, Kinyanjui H, et al. Chemotherapy-based control of schistosomiasis haematobia. II. Metrifonate vs. praziquantel in control of infection-associated morbidity. Am J Trop Med Hyg. 1990;42(6):587-95. https://doi.org/10.4269/ajtmh.1990.42.587.

149. King CH, Muchiri EM, Mungai P, Ouma JH, Kadzo H, Magak P, et al. Randomized comparison of low-dose versus standard-dose praziquantel therapy in treatment of urinary tract morbidity due to Schistosoma haema tobium infection. Am J Trop Med Hyg. 2002;66(6):725-30. https://doi.org/10.4269/ajtmh.2002.66.725.

150. Mohyelden K, Hussein HA, El Helaly HA, Ibrahem H, Abdelwahab H. Long-Term Outcomes of Two Ipsilateral vs Single Double-J Stent After Laser Endoureterotomy for Bilharzial Ureteral Strictures. J Endourol. 2020. https://doi.org/10.1089/end.2020.0956.

151. Ouma JH, King CH, Muchiri EM, Mungai P, Koech DK, Ireri E, et al. Late benefits 10-18 years after drug therapy for infection with Schistosoma haematobium in Kwale District, Coast Province, Kenya. Am J Trop Med Hyg. 2005;73(2):359-64.

152. Rasendramino MH, Rajaona HR, Ramarokoto CE, Ravaoalimalala VE, Leutscher P, Cordonnier D, et al. [Effect of praziquantel on the uro-nephrologic complications of urinary bilharziasis]. Nephrologie. 1998;19(6):347-51.

153. Ravi G, Motalib MA. Surgical correction of bilharzial ureteric stricture by Boari flap technique. Br J Urol. 1993;71(5):535-8. https://doi.org/10.1111/j.1464-410x.1993.tb16021.x.

154. Subramanian AK, Mungai P, Ouma JH, Magak P, King CH, Mahmoud AA, et al. Long-term suppression of adult bladder morbidity and severe hydronephrosis following selective population chemotherapy for Schistosoma haematobium. Am J Trop Med Hyg. 1999;61(3):476-81. https://doi.org/10.4269/ajtmh.1999.61.476.

155. Abdou A, Tligui M, Le Loup G, Raynal G. [A western cohort of urinary schistosomiasis]. Prog Urol. 2012;22(10):598-601. https://doi.org/10.1016/j.purol.2012.03.004.

156. Almeida M, Canas-Marques R, Lopez-Beltran A, Rebola J, Lúcio R, Montironi R, et al. Small cell carcinoma of the bladder associated with schistosomiasis: a case report. Anal Quant Cytopathol Histpathol. 2014;36(6):339-44.

157. Aly MS, Khaled HM, Emara M, Hussein TD. Cytogenetic profile of locally advanced and metastatic Schistosoma-related bladder cancer and response to chemotherapy. Cancer Genet. 2012;205(4):156-62. https://doi.org/10.1016/j.cancergen.2012.01.011.

158. Wishahi MM, Ismail IM, el-Sherbini M. Immunotherapy with bacille Calmette-Guérin in patients with superficial transitional cell carcinoma of the bladder associated with bilharziasis. Br J Urol. 1994;73(6):649-54. https://doi.org/10.1111/j.1464-410x.1994.tb07550.x.

159. Wishahi MM, Ismail IM, Ruebben H, Otto T. Keyhole-limpet hemocyanin immunotherapy in the bilharzial bladder: a new treatment modality? Phase II trial: superficial bladder cancer. J Urol. 1995;153(3 Pt 2):926-8.

160. Aminu MB, Abdullahi K, Dattijo LM. Tubal ectopic gestation associated with genital schistosomiasis: a case report. Afr J Reprod Health. 2014;18(2):144-6.

161. De Muylder X. Ectopic pregnancy in Zimbabwe. Int J Gynaecol Obstet. 1991;35(1):55-60. https://doi.org/10.1016/0020-7292(91)90064-c.

162. Drew LB, Tang JH, Norris A, Reese PC, Mwale M, Mataya R, et al. Schistosomiasis among obstetric fistula patients in Lilongwe, Malawi. Malawi Med J. 2018;30(4):225-9. https://doi.org/10.4314/mmj.v30i4.3.

163. Silva IM, Thiengo R, Conceição MJ, Rey L, Lenzi HL, Pereira Filho E, et al. Therapeutic failure of praziquantel in the treatment of Schistosoma haematobium infection in Brazilians returning from Africa. Mem Inst Oswaldo Cruz. 2005;100(4):445-9. https://doi.org/10.1590/s0074-02762005000400018.
